# Structural and functional insights into activation and regulation of the dynein-dynactin-NuMA complex

**DOI:** 10.1101/2024.11.26.625568

**Published:** 2024-12-03

**Authors:** Merve Aslan, Ennio A. d’Amico, Nathan H. Cho, Aryan Taheri, Yuanchang Zhao, Xinyue Zhong, Madeline Blaauw, Andrew P. Carter, Sophie Dumont, Ahmet Yildiz

**Affiliations:** 1 Biophysics Graduate Group, University of California, Berkeley USA; 2 MRC Laboratory of Molecular Biology, Cambridge United Kingdom; 3 Tetrad Graduate Program, University of California, San Francisco USA; 4 Department of Bioengineering and Therapeutic Sciences, University of California, San Francisco USA; 5 Department of Molecular and Cell Biology, University of California, Berkeley USA; 6 UCB-UCSF Bioengineering Graduate Program, San Francisco USA; 7 Department of Biochemistry & Biophysics, University of California, San Francisco USA; 8 Chan Zuckerberg Biohub, San Francisco, CA.; 9 Physics Department, University of California, Berkeley USA

## Abstract

During cell division, NuMA orchestrates the focusing of microtubule minus-ends in spindle poles and cortical force generation on astral microtubules by interacting with dynein motors, microtubules, and other cellular factors. Here we used in vitro reconstitution, cryo-electron microscopy, and live cell imaging to understand the mechanism and regulation of NuMA. We determined the structure of the processive dynein/dynactin/NuMA complex (DDN) and showed that the NuMA N-terminus drives dynein motility in vitro and facilitates dynein-mediated transport in live cells. The C-terminus of NuMA directly binds to and suppresses the dynamics of the microtubule minus-end. Full-length NuMA is autoinhibited, but mitotically phosphorylated NuMA activates dynein in vitro and interphase cells. Together with dynein, activated full-length NuMA focuses microtubule minus-ends into aster-like structures. The binding of the cortical protein LGN to the NuMA C-terminus results in preferential binding of NuMA to the microtubule plus-end. These results provide critical insights into the activation of NuMA and dynein for their functions in the spindle body and the cell cortex.

## Introduction

**Fig. 1.**
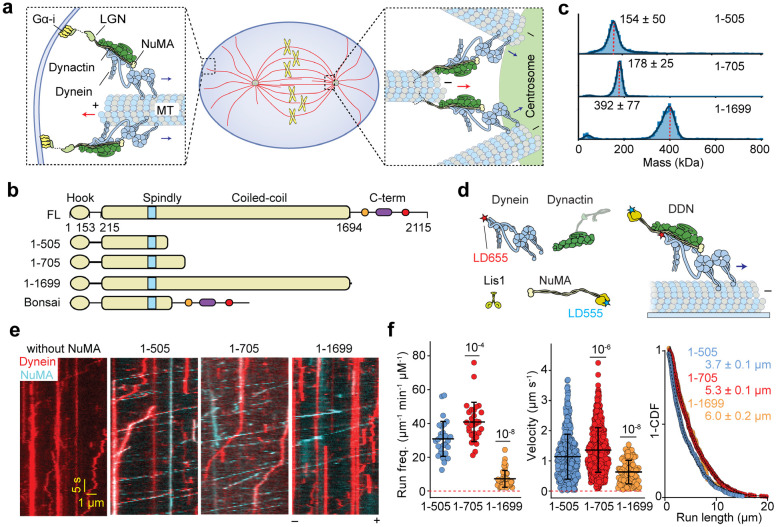
NuMA is a bona fide activator of dynein and dynactin. **a.** Schematic for the mitotic roles of NuMA. (Left) NuMA recruits dynein/dynactin to the cell cortex by interacting with Gαi subunits of heterotrimeric G proteins and LGN. DDN complexes move toward the MT minus-end (blue arrows), pull on astral MTs (red arrows) and generate tension on the mitotic spindle. (Right) In spindle poles, DDN sorts and focuses the minus-ends of MTs. **b.** Domain organization and truncations of human NuMA. The N-terminal portion of the coiled-coil domain contains the CC1 box and Spindly motif that recruits dynein and dynactin. **c.** Mass photometry shows that NuMA 1-505, 1-705, and 1-1699 form homodimers (mean ± s.d.). **d.** Schematic depiction of the *in vitro* reconstitution and single molecule imaging of DDN motility on surface-immobilized MTs. Lis1 was added to stimulate the formation of the DDN complex. Dynein and NuMA were labeled with LD655 and LD555 dyes, respectively. **e.** Kymographs show the processive motility of DDN complexes formed with different NuMA constructs *in vitro*. The assays were performed in 1 mM ATP and 100 mM KAc. **f.** The run frequency, velocity, and run length of single DDN complexes. The centerline and whiskers represent mean and s.d., respectively (From left to right, n = 29, 29, and 43 MTs for the run frequency and 418, 682, and 93 motors for the velocity and run length measurements). *P* values are calculated from a two-tailed t-test. The inverse cumulative distribution functions (1-CDF) of motor run length were fit to a single exponential decay to determine the mean run length (±s.e.).

Nuclear mitotic apparatus protein (NuMA) interacts with DNA, microtubules (MTs), motors, plasma membranes, importins, and other accessory proteins for fundamental cellular processes^1^. In interphase, NuMA localizes to the nucleus, where it organizes chromosomes and helps the formation of a single, round nucleus^2, 3^. After nuclear envelope breakdown, NuMA accumulates at MT minus-ends and recruits a minus-end-directed MT motor, dynein. Together with dynein, NuMA helps focus the minus-ends of the MTs into spindle poles and maintain a steady-state spindle structure^4-9^ (Fig. 1a). Minus-end-directed forces generated by dynein counteract forces generated by plus-end-directed kinesin-5s^10, 11^ to maintain spindle structure^12, 13^. In anaphase, a population of NuMA dissociates from the spindle poles, moves to the cell cortex, and interacts with the scaffold protein LGN to form the NuMA/LGN/Gαi complex at the cell cortex^14^ (Fig. 1a). Cortical NuMA recruits dynein, which anchors astral MTs and generates tension to determine the position and orientation of the spindle^15-17^. Consistent with its cellular roles, NuMA dysfunction causes defects in spindle mechanics and architecture, as well as the micronuclei formation^1^.

NuMA is a large (237 kDa) and extended protein containing an N-terminal globular HOOK domain (amino acids (a.a.) 1-153) and a C-terminal region (a.a. 1700-2115) separated by a ~210 nm long long coiled-coil domain^18, 19^. The central coiled-coil facilitates dimerization^20^ and prevents the binding of NuMA to chromosomes during mitosis^3^. The coiled-coil also provides the extension needed for properly crosslinking MTs and DNA^3, 21, 22^ as well as efficiently capturing astral MTs at the cell cortex^23^. The C-terminal region contains two MT-binding domains (MTBD1 (a.a. 1914-1985) and MTBD2 (a.a. 2002-2115)), a clustering motif (a.a. 1768-1777), nuclear localization signal (NLS; a.a. 1988-2005), LGN binding site (a.a. 1900-1926), and DNA binding site (a.a. 2058-2115)^1^. MTBD2 can bind to the MT lattice^24^ and is required for spindle-pulling activity at the cortex in human cells^17^. MTBD1 has a weaker affinity for MTs^25^, but it is required for spindle pole focusing^26, 27^ and accumulation of NuMA to the minus-end of MTs^9^ in mammalian cells. There are also contradictory reports for the distinct roles of MTBD1 and MTBD2 in spindle pole focusing and orientation in other cell types^28^, and it remains unclear how these MTBDs bind MTs and regulate MT dynamics. MTBD1 and MTBD2 sites partially overlap with LGN and DNA binding sites, respectively, suggesting that the association of NuMA with other interaction partners affects its MT binding^29^.

Dynein forms a 1.4 MDa complex consisting of two copies of six subunits (heavy, intermediate, and light intermediate chains, and three light chains, Fig.1a)^30^. The dynein heavy chain is the largest subunit, which contains AAA+ ATPase and MT binding domains. The tail of the heavy chain facilitates homodimerization and recruitment of other subunits. Isolated dynein is autoinhibited and does not actively move along the MT^31, 32^. Activation of dynein motility requires recruitment to its cofactor dynactin (a 23-subunit, 1.0 MDa complex) via the coiled-coil region of adaptor proteins^33-35^. These activating adaptors contain several conserved motifs such as a specific binding site (i.e., CC1-box, HOOK, and EF-hand domains) for the C terminus of the dynein light intermediate chain (DLIC), a heavy chain binding site 1 (HBS1) motif for interaction with the dynein heavy chain (DHC) and a Spindly motif for interacting with the pointed end subcomplex of dynactin^36^. Studies in live cells showed that the N-terminal portion of NuMA (a.a. 1-505) is required for the recruitment of dynein to the cell cortex^17^. This region of NuMA contains a coiled coil and two regions reported to interact with the DLIC (an N-terminal HOOK domain and a CC1-box-like motif (a.a. 360–385))^37^, along with a predicted Spindly motif (a.a. 417–422)^17^ (Fig. 1b). Collectively, these observations suggest that the N-terminus of NuMA can in principle form a complex with dynein and its cofactor dynactin. However, the presence of two distinct DLIC-binding sites, which has not been observed in any other activating adaptor, as well as the absence of a clear HBS1, limit our understanding of how NuMA interacts with dynein and dynactin. It also remains unclear whether NuMA requires additional factors or post-translational modifications to activate dynein.

Our understanding of how NuMA orchestrates spindle assembly, maintenance, and orientation mostly comes from studies in cells. The molecular mechanisms by which NuMA recruits dynein/dynactin, localizes to the minus-ends of spindle MTs, focuses MT minus-ends together, and interacts with LGN to facilitate cortical force generation, remain unclear. How NuMA is regulated and repurposed for its mitotic functions is also not well understood. The absence of in-depth *in vitro* biochemical and biophysical characterization of NuMA was partly due to its large size, low solubility, and tendency to form large clusters^17, 19, 38^. In this study, we used biochemical reconstitution, cryo-electron microscopy (cryo-EM), *in vitro* motility assays, and live cell imaging to investigate how human NuMA binds MTs and focuses MTs together with dynein. We demonstrated that the NuMA N-terminus forms a ternary complex with dynein and dynactin and activates dynein motility, whereas the C-terminal region preferentially binds and suppresses the dynamicity of the minus-end of MTs. Full-length (FL) NuMA only weakly binds MTs or activates dynein, whereas mitotically phosphorylated NuMA activates dynein motility in vitro and triggers dynein-driven transport in interphase cells. Together with dynein, active FL NuMA focuses minus-ends of MTs into aster-like structures *in vitro*. LGN binding to the NuMA C-terminus shifts the preferential binding of NuMA to the MT plus end, consistent with the interaction of NuMA with the plus-ends of astral MTs at the cell cortex. These results provide key insights into how NuMA organizes MTs and drives mitotic functions of dynein in the spindle body and at the cell cortex.

## Results

### The NuMA N-terminus assembles a DDN complex and activates dynein motility

**Extended Data Figure 1.**
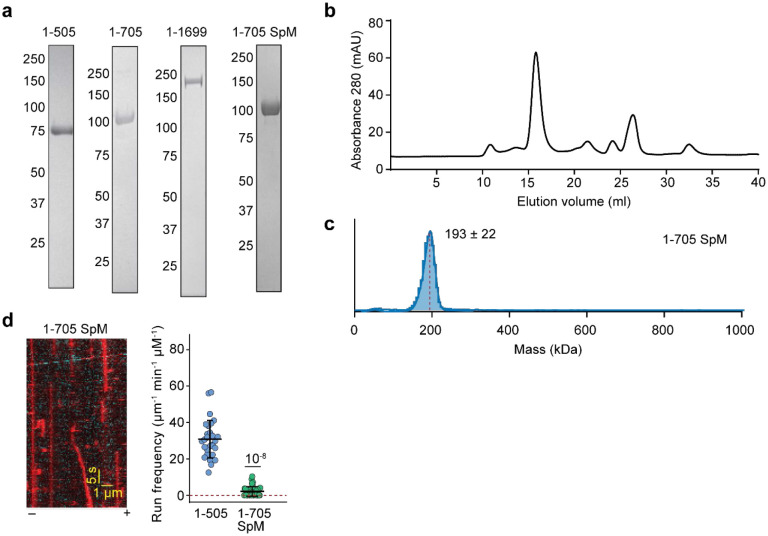
Biochemical characterization and motility of N-terminal NuMA constructs. **a.** Denaturing gel pictures of N-terminal NuMA constructs after gel filtration. The numbers on the left represent molecular weight in kDa. **b.** Elution of NuMA 1-505 from a Superose 6 gel filtration column. **c.** Mass photometry shows that NuMA 1-705 SpM forms a homodimer (mean ± s.d.). **d.** (Left) An example kymograph of DDN complexes formed with NuMA 1-705 SpM *in vitro*. Dynein and NuMA are colored with red and cyan, respectively. The assay was performed in 1 mM ATP and 100 mM KAc. (Right) The comparison of the run frequencies of single DDN complexes assembled with NuMA 1-505 and NuMA 1-705 SpM. The centerline and whiskers represent mean and s.d., respectively (From left to right, n = 29 and 40 MTs). The *P* value is calculated from a two-tailed t-test. The velocity and run length of complexes formed with NuMA 1-705 SpM could not be determined due to the infrequency of processive runs.

To test the ability of NuMA to form a ternary complex with purified dynein-dynactin and to activate dynein motility *in vitro*, we recombinantly expressed and purified three human NuMA constructs containing the N-terminal coiled-coil (1-505, 1-705, and 1-1699) from insect cells (Fig. 1b, Extended Data Fig. 1). Mass photometry showed that all three constructs form homodimers (Fig. 1c)^20, 39^. We next performed multi-color single-molecule motility assays on surface-immobilized taxol-stabilized MTs in the presence of dynein, dynactin, and the dynein regulator Lis1 (Fig. 1d)^40, 41^. In the absence of NuMA, dynein did not exhibit any processive runs on MTs. The addition of NuMA N-terminal constructs resulted in the activation of dynein motility and comigration of NuMA with dynein towards the minus-ends of MTs (Fig. 1e, Supplementary Movie 1). The velocity, run length, and run frequency of dynein/dynactin/NuMA (DDN) complexes with shorter N-terminal coiled coils were comparable to dynein-dynactin complexes with other activating adaptors, such as BICDR1^42^ (Fig. 1f). However, DDN with NuMA 1-1699 had ~5-fold lower run frequency and ~30% lower velocity than that of the shorter NuMA fragments, suggesting that the long coiled-coil has a partial inhibitory effect on DDN motility. These results showed that NuMA is a bona fide cargo adaptor that activates dynein-dynactin motility.

### Cryo-EM structure of the DDNL complex

**Extended Data Figure 2.**
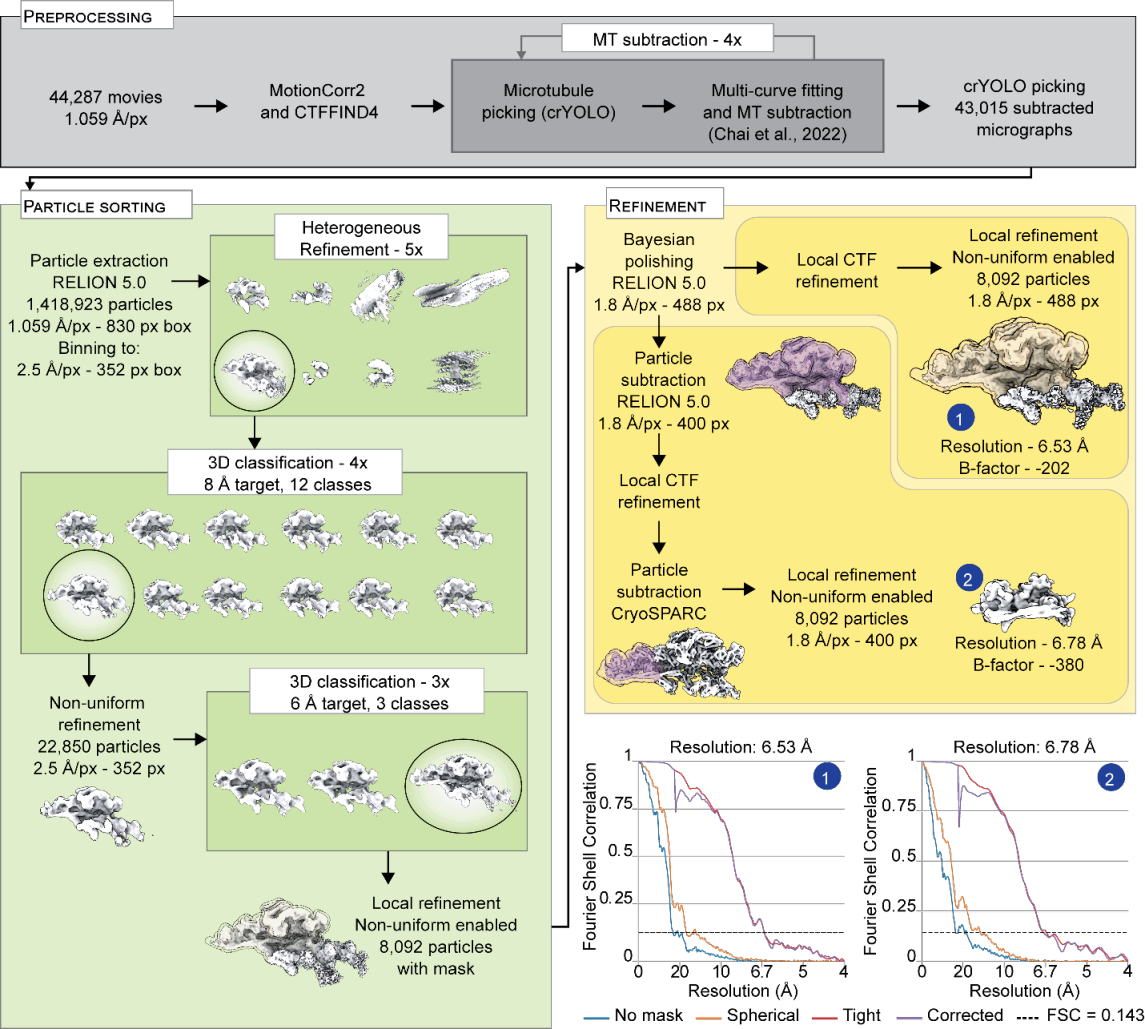
Cryo-EM image processing pipeline for DDN complexes in the presence of Lis1. All refinements and classifications were performed in CryoSPARC unless otherwise specified. The masks used for particle subtraction are displayed in purple. Static masks used for refinements are displayed in yellow. When no mask is displayed, a dynamic mask was used instead. Before contrast transfer function (CTF) refinement, a non-uniform refinement was performed (not shown). The plots show gold-standard Fourier shell correlation for the final maps.

**Extended Data Figure 3.**
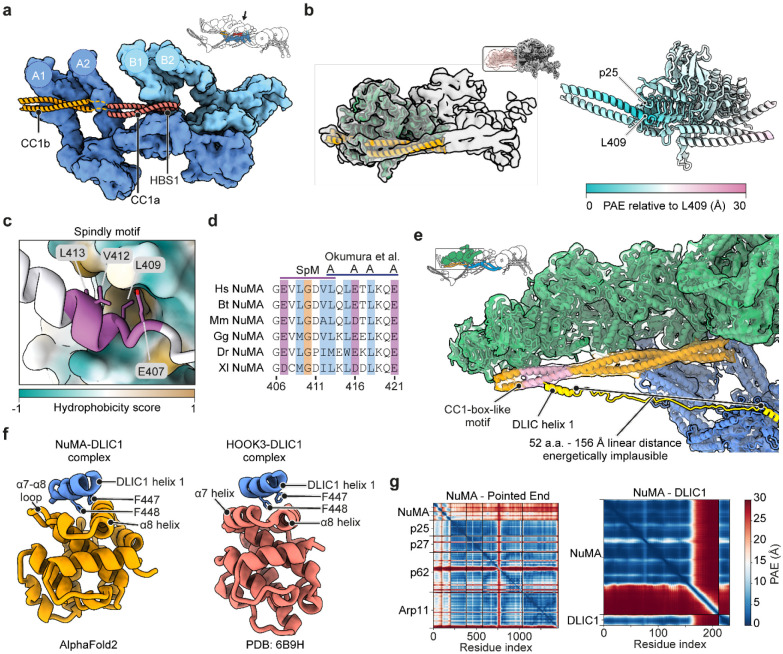
Structural analysis of the interactions between NuMA and dynein-dynactin. **a.** The organization of the dynein-A and dynein-B interactions with the NuMA coiled-coil, with the dynactin subunits hidden. The arrow in the insert shows the viewing angle of the DDN complex. **b.** Fitting of an AF2 model of NuMA 351-450 bound to the dynactin pointed end complex into a locally refined EM density for the same region of dynactin, with the mask used and model colored by local predicted alignment error (PAE) values. Residues with low predicted local distance difference test (pLDDT) scores are hidden. **c.** The Spindly motif of NuMA bound to the p25 subunit of dynactin, colored by hydrophobicity. **d.** Sequence alignment of the Spindly motif region in NuMA orthologues, with the location of the mutations from Okumura et al.^17^. **e.** Close-up view of the CC1-box-like motif (pink) on NuMA in our EM density. A model of the DLIC tail with the unstructured region extended illustrates that the interaction is likely unfavored due to the distance. **f.** Comparison between the AF2 model of the NuMA Hook domain interaction with DLIC1 and the published structure of the complex between the HOOK3 hook domain and DLIC1 (PDB 6B9H)^47^. Residues outside of the HOOK domain and DLIC are not displayed. **g.** PAE plots for AF2 complex models in a and f.

**Fig. 2.**
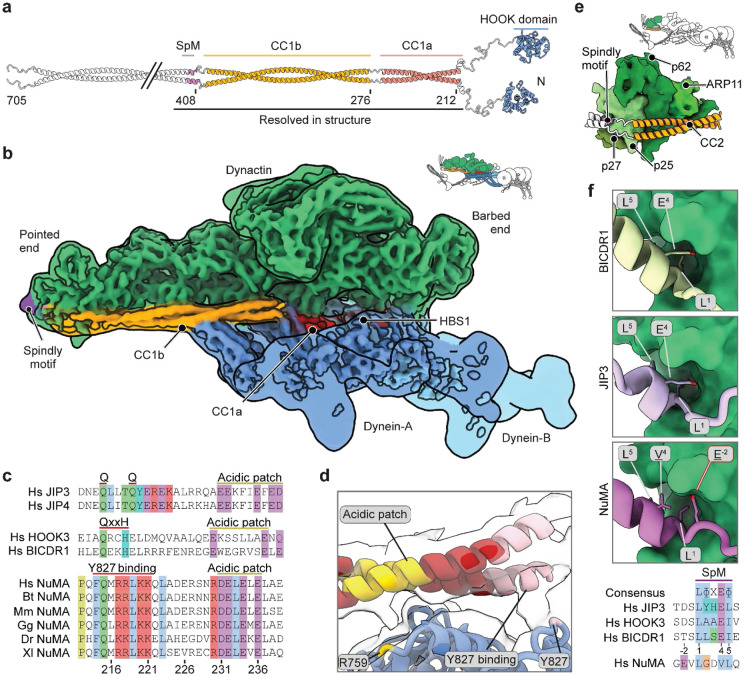
Cryo-EM structure of dynein-dynactin-NuMA-Lis1 on MTs. **a.** Linearized AF2 prediction of NuMA 1-705, the construct used for this structure. **b.** EM density map of dynein-dynactin bound to NuMA and Lis1. The dynein motor domains, p150 arm, and Lis1 are not resolved. **c.** Multiple sequence alignment of HBS1 from NuMA orthologues. Other activating adaptors with known HBS1 sequences are shown for comparison. **d.** Cryo-EM density and model of the NuMA HBS1-DHC interfaces. **e.** AF2 model of the NuMA interaction with the dynactin pointed end. Individual dynactin subunits are labeled. **f**. Spindly motif interactions with the dynactin pointed end for JIP3 (PDB: 8PR4), BICDR1 (PDB: 7Z8M), and NuMA (AF2, this study) illustrate the different positions of the interacting residues, with multiple sequence alignment of the Spindly motif of the adaptors JIP3, HOOK3, BICDR1 and NuMA.

To understand how NuMA interacts with dynein and dynactin, we prepared DDN complexes with NuMA 1-705 in the presence of Lis1 and used a previously established cryo-EM workflow^43, 44^ to obtain a ~6.5 Å resolution map of the complex on MTs (Fig. 2a-b; Extended Data Fig. 2). NuMA coiled-coil segments run along the length of dynactin, extending to the pointed end, and recruit two dynein motors (dynein-A and -B, Fig. 2b). The AlphaFold2 (AF2) model of NuMA predicts a break in the first coiled-coil between CC1a (a.a. 212-269) and CC1b (a.a. 276-396) that agrees with the density in our map (Fig. 2b, Extended Data Fig. 3a). Our map also shows extra density visible outside the pointed end. Based on a high-confidence AF2 model of the interaction between NuMA and the dynactin pointed end (Extended Data Fig. 3b), we assigned this density to the Spindly motif site at the N-terminus of CC2 (408-705; Fig. 2a-b).

In our model, CC1a contacts dynein-A at a site that includes DHC residues Y827 and R759. In other dynein/dynactin complexes^43, 44^, these residues bind the adaptor HBS1 motif, which consists of a conserved Gln residue and acidic patch (Fig 2c-d). AF2 is unable to predict a NuMA HBS1-DHC interaction. However, from the position of the CC1a fragment, NuMA is likely to bind dynein-A using an acidic patch (residues 231-240) and a conserved region near Q215 (Fig. 2d). Although the HBS1 site is typically located in the middle of the coiled-coil in other activating adaptors^44^, this putative HBS1 site of NuMA is at the very N-terminus of the coiled-coil marked by a conserved proline residue (P212).

NuMA makes multiple contacts with the pointed end subunits of dynactin through both CC1b and the Spindly motif on CC2 (Fig. 2e), but its Spindly motif differs from the consensus LxxEΦ (where Φ is hydrophobic) found in other adaptors^44^. In BICDR1 and JIP3, the hydrophobic residues in the sequence bind a pocket on the pointed-end p25 subunit, while the glutamate shields the pocket and interacts with N20 on p25 (Fig. 2f). In NuMA, the model predicts the Spindly motif sequence is LGDVL. The two leucines (L409 and L413) bind the hydrophobic pocket on p25, whereas a glutamate outside the core motif (E407) contacts N20 (Fig. 2f; Extended Data Fig. 3c). A previously suggested position for the Spindly motif^17^ is downstream from the predicted sequence but overlaps with it at residue L413. This may explain why alanine substitutions to the previously reported Spindly mutant impair dynein activity both in cellular assays^17^ and in our *in vitro* assays (NuMA 1-705 SpM, Extended Data Figs. 1e and 3d), consistent with the requirement of this motif in the activation of dynein motility by other adaptor proteins^45, 46^.

NuMA is reported to bind the C-terminus of DLIC both through its Hook domain and a CC1-box-like motif^37^. In our structure, the proposed CC1-box-like motif sits on the far end of CC1b, in contact with the dynactin pointed end. Distance constraints and the lack of any additional density corresponding to the DLIC indicate that the CC1-box-like motif is unlikely to be engaged in interactions with dynein in our structure (Extended Data Fig. 3e). While we cannot resolve the NuMA Hook domain due to its flexibility, an AF2 prediction suggests the DLIC1 helix, with its two conserved phenylalanines fits between the α8 helix and the α7-α8 loop of the NuMA Hook domain^37^ (Extended Data Fig. 3f). This position is consistent with previous mutational analysis^37^. Compared to the structure of the HOOK3 Hook domain – DLIC1 helix complex^47^, the NuMA Hook domain lacks an α7 helix, which translates to closer contact with the α8 helix. Collectively, our structural analysis showed that NuMA binds dynein and dynactin through an HBS1 site and a previously unknown Spindly motif, and that a proposed CC1-box-like motif is not likely involved in complex formation.

### MTBD1 Preferentially Binds the Minus-end of MTs

**Extended Data Figure 4.**
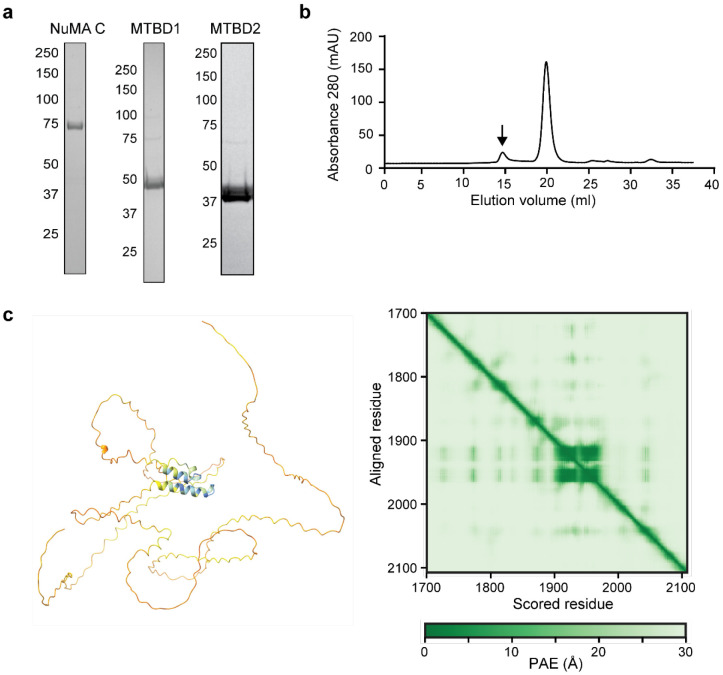
Biochemical characterization and structure prediction of C-terminal NuMA constructs. **a.** Denaturing gel pictures of C-terminal NuMA constructs after gel filtration. **b.** The elution profile of NuMA C (black arrow) from a Superdex 200 gel filtration column. **c.** (Left) AF2 predicts that the NuMA C-terminus is almost fully unstructured except for a single short helix in MTBD1. (Right) PAE for the model on the left.

**Extended Data Figure 5.**
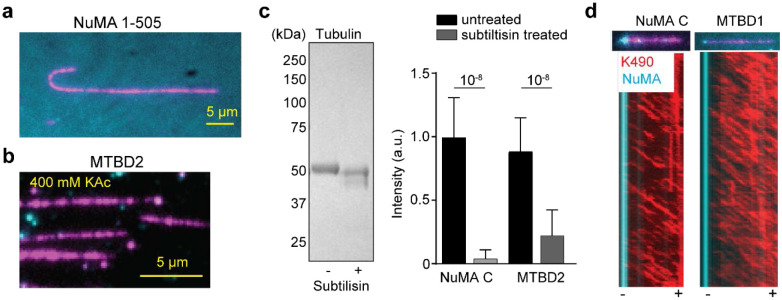
MT binding of C-terminal NuMA constructs. **a.** An example picture shows that NuMA 1-505 (cyan) in the flow chamber does not bind to MTs (magenta). **b.** An example picture shows that MTBD2 (cyan) does not exhibit any detectable MT (magenta) binding when the salt concentration is increased to 400 mM. **c.** (Left) The denaturing gel picture shows the reduction of the molecular weight of tubulin due to the cleavage of tubulin C-terminal tails by subtilisin treatment. (Right) The normalized fluorescence intensity of 300 nM NuMA C and 50 nM MTBD2 on surface-immobilized MTs in the presence and absence of subtilisin treatment. Error bars represent s.d. (n = 119, 166, 70, and 174 MTs from left to right). P-values are calculated from a two-tailed t-test. **d.** Two color kymographs of K490 (red) and NuMA C or MTBD1 (cyan) on the MT. MT polarity was determined from the plus-end directed motility of K490 motors. NuMA accumulates at the minus-end of the MTs.

**Fig. 3.**
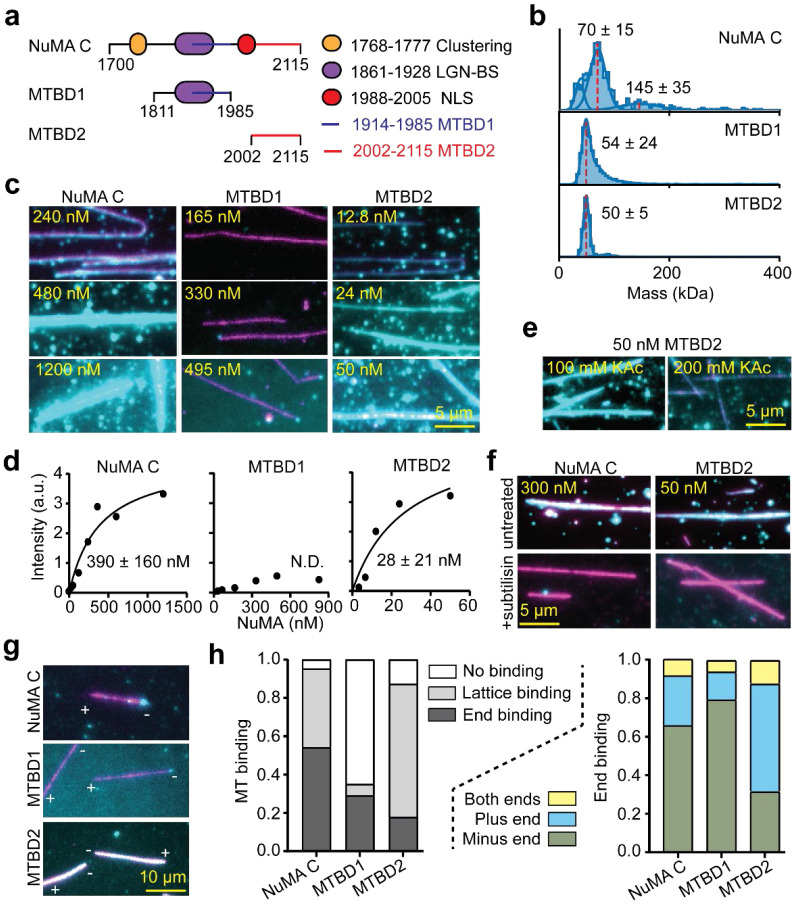
The C-terminus of NuMA preferentially binds to the minus-end of MTs. **a.** The NuMA C-terminal region contains two MTBDs (MTBD1 and 2), a clustering motif, an LGN binding site (LGN-BS), and a nuclear localization signal (NLS). **b.** Mass photometry shows that the C-terminal constructs primarily form monomers (mean ±s.d.). **c**. Example pictures show NuMA C and MTBD2, but not MTBD1, densely decorated surface-immobilized MTs. **d**. The fluorescence intensity of NuMA constructs per length of an MT (n = 47, 46, 48, 53, 48, 48, 61, 18, 50, 52, 50, 54, 43, 59, 39, 62, 62, 60, 63, and 75 MTs from left to right). K_d_ values are calculated from a fit to a binding isotherm (solid black curves; ±s.e.; N.D.: not determined). **e.** MT binding of MTBD2 is greatly reduced by increasing the salt concentration. **f.** NuMA C and MTBD2 exhibit little to no binding to subtilisin-treated MTs. **g.** Example pictures show MT binding of C-terminal constructs at concentrations lower than their respective K_d_ values. MT polarity is determined from the directionality of plus-end-directed Cy5-labeled K490 motors (not shown). **h.** Normalized MT binding and end binding preference of C-terminal constructs (n = 107, 117, and 142 MTs from left to right). In c, e, f, and g, NuMA and MTs are colored in cyan and magenta, respectively.

To understand the interaction of the NuMA C-terminus with MTs, we purified NuMA C (a.a. 1700 - 2115), MTBD1 (a.a. 1811 – 1985; previously referred to as NuMA TIP^28^), and MTBD2 (a.a. 2002 – 2115; Fig. 3a, Extended Data Fig. 4a-b). Although earlier studies referred to the NuMA C-terminus as a globular domain^48, 49^, AF2 predicts that it is almost fully disordered (Extended Data Fig. 4c), consistent with the interaction of this region with a diverse array of cellular factors and the liquid-liquid phase separation of NuMA both *in vitro* and *in vivo*^38^. The C-terminal constructs lack the coiled-coil region and primarily appear as monomers in mass photometry (Fig. 3b). We found that NuMA C, which contains both MTBD1 and MTBD2 sites, appeared both on the lattice and the tip of MTs (Fig. 3c-d). Consistent with a previous report^25^, MTBD1 weakly interacted with the MT lattice. However, it mostly appeared as bright puncta at the tip of a subset of MTs. MTBD2 densely decorated the MT lattice with an apparent dissociation constant (K_d_) of 28 nM. Surprisingly, the MT affinity of NuMA C was substantially lower than that of MTBD2 (390 nM, Fig 3c-d), suggesting that self-interactions within the NuMA C-terminus restrict the interaction of its MTBDs with MTs. MT association of MTBD2 weakened upon increasing the salt concentration to 200 mM KAc (Fig. 3e, Extended Data Fig. 5), suggesting electrostatic interactions between NuMA and MTs. The interaction of NuMA C and MTBD2 with MTs was also substantially reduced when the tubulin tails were cleaved by a serine protease, subtilisin, indicating that tubulin tails play an important role in MT binding of NuMA (Fig. 3f, Extended Data Fig. 5c).

Previous studies in live cells hinted that MTBD1 is sufficient for binding of NuMA to minus-ends of MTs, whereas MTBD1 or MTBD2 prefers curled ends of MTs and is required for localization of NuMA at the plus-end tip of astral MTs^9, 24, 28, 50^. We quantified the lattice and tip binding preference NuMA C, MTBD1, and MTBD2 at concentrations lower than their respective K_d_ values (Fig. 3g,h, Extended Data Fig. 5d). MTBD1 rarely binds to the lattice and strongly (~80%) prefers to bind to the minus-end when it lands on the MT. MTBD2 decorates the lattice even at low concentrations and does not exhibit strong tip binding (~20%), but it prefers to land on the plus-end when it only appears at the MT end. NuMA C exhibits the MT binding features of both MTBD1 and MTBD2, appearing both at the ends and the lattice of MTs, but prefers the minus-end when it only appears at the tip of the MT (Fig. 3g-h). These results show that NuMA can recognize and bind to the minus-ends of MTs through MTBD1^9^, but it also interacts with the lattice and the plus-end through MTBD2^24, 50^.

### NuMA is Rescued from Autoinhibition by Phosphorylation of its C-terminus

**Extended Data Figure 6.**
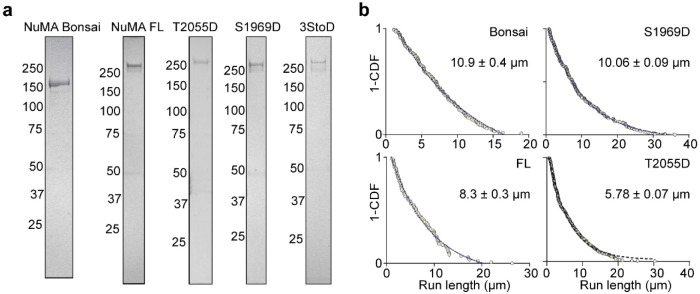
Expression of NuMA Bonsai and NuMA FL constructs. **a.** Denaturing gel pictures of NuMA FL and Bonsai constructs after gel filtration. **b.** The run lengths of single DDN complexes assembled with NuMA FL and Bonsai constructs. (n = 154, 87, 243, and 377 MTs motors for NuMA Bonsai, FL, S1969D, and T2055D, respectively). 1-CDFs of motor run length were fitted to a single exponential decay (blue dashed curves) to determine the mean run length (±s.e.).

**Fig. 4.**
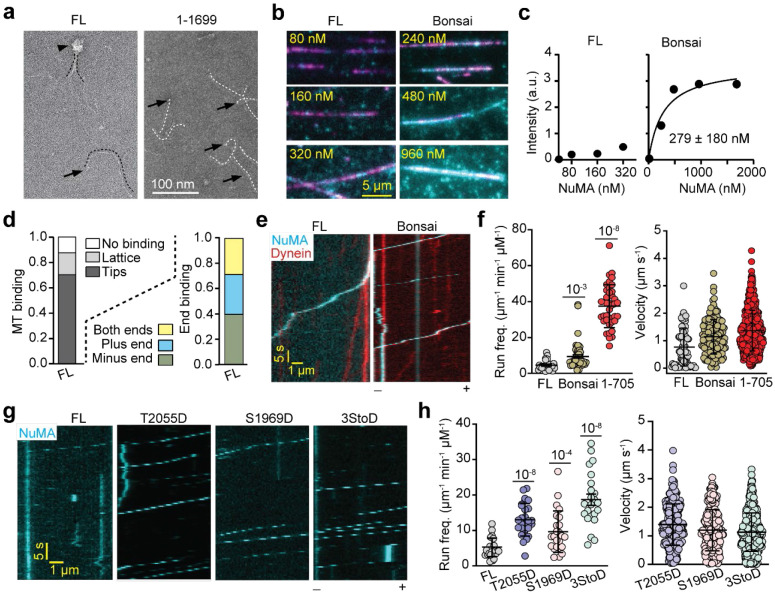
NuMA-dynein interaction is activated by the phosphorylation of its C-terminus. **a.** Negative-stain EM micrographs show that NuMA FL forms both elongated coiled coil (black arrow) and large clusters with coiled-coils pointing outward (black arrowhead), whereas NuMA 1-1699 forms only elongated coiled-coils. Dashed curves represent the trajectories of the coiled-coils. **b.** Example pictures show MT (magenta) binding of NuMA FL and Bonsai (cyan) under different concentrations. **c**. The intensity of NuMA constructs fused to mNeonGreen (mNG)per length of an MT (n = 25, 67, 67, 116, 25, 70, 25, 46, and 17 MTs from left to right). The K_d_ value of NuMA Bonsai was calculated from a fit to a binding isotherm (solid black curves; ±s.e.). **d.** Normalized MT binding and end binding preference of NuMA FL (n = 104 MTs). **e**. Kymographs show processive motility of DDN complexes assembled with NuMA FL and Bonsai. **f**. The run frequency and velocity of DDN complexes assembled with NuMA FL and Bonsai, compared to NuMA 1-705 (from left to right, n = 24, 52, 38 MTs for the run frequency and 85, 154, 682 motors for the velocity measurements). **g.** Kymographs show the processive motility of DDN complexes assembled with phosphomimetic mutants of NuMA FL. **h.** The run frequency and velocity of DDN complexes assembled with phosphomimetic mutants of NuMA FL (from left to right, n = 24, 28, 24, 23 MTs for run frequency and 286, 243, 435 motors for velocity measurements). In f and h, the centerline and whiskers represent mean and s.d., respectively. *P* values are calculated from a two-tailed t-test.

We next expressed full-length (FL) NuMA and tested its ability to bind MTs and activate dynein (Extended Data Fig. 6a). Consistent with previous reports^19, 39^, negative stain EM imaging revealed that NuMA FL forms large clusters projecting ~200 nm long and flexible extension arms (Fig. 4a). In contrast, NuMA 1-1699 only forms ~200 nm long extension arms without clusters (Fig. 4a), demonstrating that the C-terminus is responsible for the clustering of NuMA FL and long extension arms correspond to the coiled-coil domain. Clustering of NuMA FL was also observed in fluorescence imaging assays (Fig. 4b). These observations are in line with the punctate signals of NuMA at the cell cortex^17^ and the proposed function of NuMA in crosslinking and focusing MT minus-ends into larger asters in acentrosomal spindles^50^.

NuMA FL had a low affinity for MTs and exhibited no clear preference for tip binding (Fig. 4b-d). NuMA FL also resulted in ~10-fold less frequent processive runs and ~2-fold slower movement than complexes assembled with NuMA 1-705 under the same conditions (Fig. 4e-f, Extended Data Fig. 6b). In comparison, NuMA Bonsai, which contains both the N-terminal (1-705) and C-terminal (1700-2115) region but lacks the intervening coiled-coil domain^3, 17^, binds to MTs more efficiently than NuMA FL, with a similar MT affinity of NuMA C (Fig. 4b-d). NuMA Bonsai also resulted in more frequent dynein runs than NuMA FL, albeit at a lower frequency than NuMA 1-705 (Fig. 4e-f). DDN Bonsai moved at a similar velocity to that of complexes assembled with NuMA 1-705 and 1-505 (Fig. 4f), suggesting that the presence of NuMA MTBDs does not cause intermittent pausing or dragging against dynein motility. These results show that NuMA FL is partially autoinhibited for its mitotic functions. Because removal of the central coiled-coil partially rescues MT binding and dynein activation, NuMA autoinhibition may involve interactions between the N- and C-terminal regions, analogous to other cargo adaptors of dynein^51-53^.

**Extended Data Figure 7.**
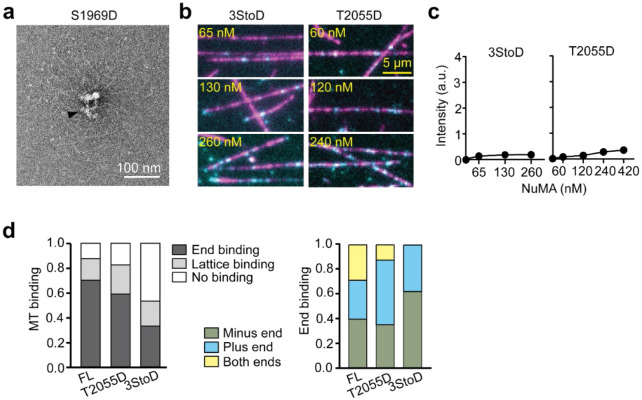
MT binding of phosphomimetic mutants of NuMA. **a.** An example negative-stain EM micrograph shows that NuMA S1969D forms a large cluster with coiled-coils pointing outward (black arrowhead). **b.** Example pictures show MT (magenta) binding of NuMA 3StoD and T2055D (cyan) under different concentrations. **c**. The intensity of NuMA 3StoD and T2055D per length of an MT (n = 25, 98, 101, 101, 25, 120, 131, 116, and 112 MTs from left to right). **d.** Normalized MT binding and end binding preference of NuMA FL, 3StoD, and T2055D (n = 104, 150, and 178 MTs from left to right).

We reasoned that NuMA may be rescued from autoinhibition and activated for its mitotic functions through phosphorylation^54, 55^. Studies in live cells revealed that, after nuclear envelope breakdown, NuMA is phosphorylated by CDK1 at T2055 to enhance its MT binding and inhibit its localization to the cell cortex before anaphase^56, 57^. Conversely, S1969 phosphorylation by Aurora-A kinase facilitates the translocation of NuMA from spindle poles to the cell cortex at the onset of anaphase^58^. We generated phosphomimetic mutants of the Aurora A (S1969D, S1991D, and S2047D; 3StoD hereafter)^54, 58^ and CDK1 (T2055D)^56^ phosphorylation sites and tested how these modifications affect dynein activation of NuMA FL. These mutants exhibited similar MT binding properties to wild-type NuMA FL (Extended Data Fig. 7), suggesting that phosphorylation of NuMA at these sites does not substantially affect its MT binding. However, all three mutants resulted in up to a 4-fold increase in the run frequency of DDN complexes (Fig. 4g-h, Supplementary Movie 2). The velocity of these complexes was comparable, but their run frequency was 40% lower than DDN complexes assembled with NuMA 1-705 (Fig. 4f, h). These results demonstrate that interphase NuMA is autoinhibited for its interactions with dynein and activated by mitotic phosphorylation of its C-terminus by Aurora A and CDK1.

### Mitotically Phosphorylated NuMA Activates Dynein in Interphase Cells

**Figure 5.**
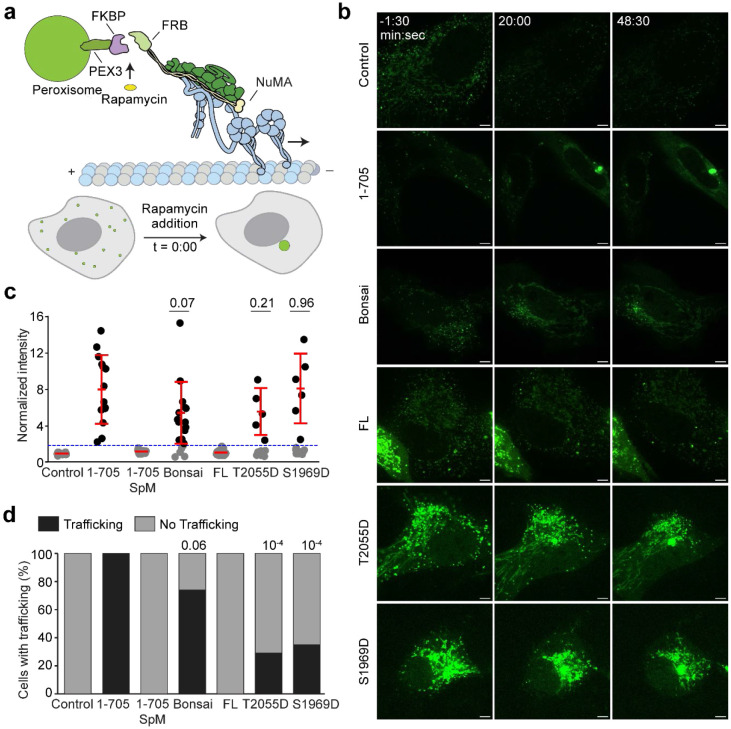
Mitotically-phosphorylated NuMA activates dynein in interphase cells. **a.** Cartoon depiction of peroxisome trafficking assay. Rapamycin addition (time 0:00) induces recruitment of a NuMA construct to peroxisomes and facilitates the trafficking of peroxisomes towards the centrosome if the NuMA construct activates dynein. **b.** Representative confocal images of peroxisomes (PEX3, green) before and after rapamycin addition in WT RPE1 cells (control) and RPE1 cells expressing different NuMA constructs. PEX3-mEmerald-FKBP levels vary between cells due to variations in transfections. Scale bar = 5 μm. **c.** Normalized mEmerald intensity at the centrosome, as assessed 45 min after rapamycin addition. Cells were scored as trafficking (black) if normalized fluorescence intensity was above the threshold (blue dashed line), which is two-fold higher than cytoplasmic background (grey; n = 16, 14, 15, 10, 22, 17, and 17 from left to right; two independent experiments). Error bars represent mean ±s.d. of trafficking cells. **d.** Percent of cells with peroxisome trafficking, from same samples as c and defined from normalized mEmerald intensity. In c and d, *P* values are calculated from a two-tailed t-test and Fisher’s exact test, respectively, in comparison to the 1-705 condition.

To investigate whether NuMA can activate dynein in cells, we leveraged synthetic activation of dynein in peroxisome transport through chemically inducible dimerization of FRB and FKBP tags upon rapamycin addition (Fig. 5a)^59^. Peroxisomes typically diffuse freely through the interphase cytoplasm. We exogenously expressed a peroxisome marker (PEX3) fused with mEmerald and FKBP and fused FRB to different NuMA constructs that lack the NLS motif. If the NuMA construct activates dynein-mediated transport, we expect PEX3-mEmerald to move to the centrosome where minus-ends terminate, typically in the perinuclear region.

**Extended Data Figure 8.**
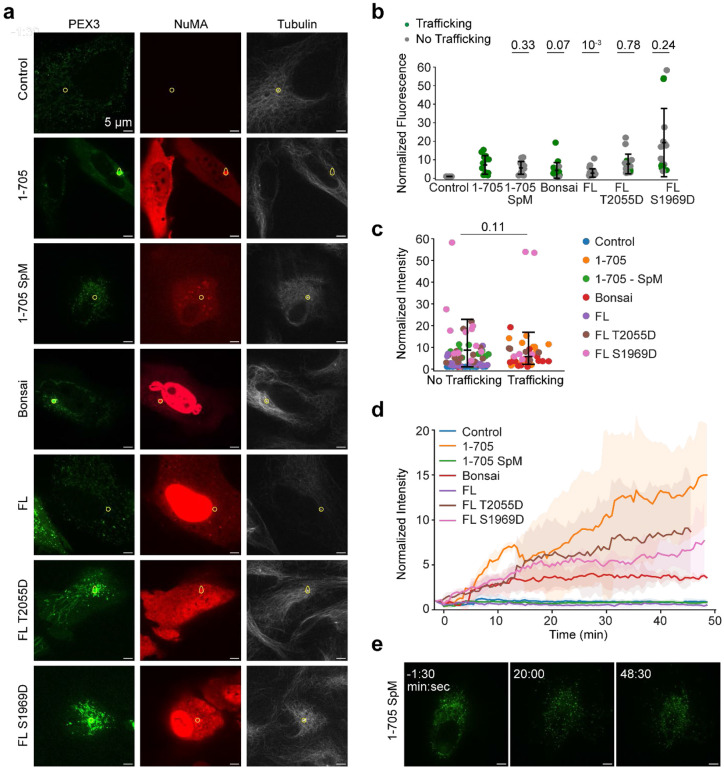
The analysis of peroxisome trafficking assays. **a.** Representative confocal images of peroxisomes (PEX3, green), NuMA (red), and tubulin (SiR-tubulin, grey) from z-stacks taken 45 minutes after rapamycin addition in WT RPE1 cells (control) and RPE1 cells expressing different NuMA constructs. Centrosomes (white dots in tubulin channel) and consequently area of quantification is marked by the yellow circle in each still. Cells are the same as in Fig. 5b. Scale bar = 5 μm. **b.** Normalized cytoplasmic NuMA concentration was calculated by averaging the mean pixel intensity for NuMA in the cytoplasm from the timepoints before rapamycin addition (n=16, 14, 15, 19, 22, 17, and 17 from left to right, two independent experiments). **c.** Normalized cytoplasmic NuMA concentration sorted by trafficking outcome. Cells are the same as in b. **d.** Average peroxisome signal accumulation over time using normalized GFP intensity, from a subset of cells where the centrosome (marked by SiR-tubulin) stayed in focus for the majority of frames. For NuMA 1-705, Bonsai, T2055D, and S1969D, only cells that had trafficking were tracked. Time points where the centrosome was out of focus were excluded. Shading represents s.d. From top to bottom, n = 7, 4, 5, 4, 2, 3, and 3 cells pooled from two independent experiments. **e.** Representative confocal images of peroxisomes (PEX3, green) before and after rapamycin addition in RPE1 cells expressing NuMA 1-705 SpM. Scale bar = 5 μm. In b and c, the centerline and whiskers represent mean and s.d., respectively. *P* values are calculated from a two-tailed t-test.

In agreement with *in vitro* results (Fig. 1), we found that NuMA 1-705 efficiently traffics peroxisomes to the centrosome in interphase cells (Fig. 5b-d, Extended Data Fig. 8, Supplementary Movie 3). A construct containing the previously predicted Spindly mutation (NuMA 1-705 SpM)^17^ fully eliminated trafficking, verifying that trafficking results specifically from NuMA-dynein interactions, as opposed to an indirect mechanism. NuMA-Bonsai, which additionally has NuMA’s C-terminus that can bind MTs, also trafficked peroxisomes. Notably, NuMA FL did not cause peroxisomes to cluster. In comparison, phosphomimetic NuMA FL constructs (S1969D and T2055D) trafficked peroxisomes similar to NuMA 1-705. These results are consistent with *in vitro* motility assays (Fig. 4) and show that NuMA can activate dynein in interphase cells when in a mitotic phosphorylation state. We concluded that NuMA autoinhibition is physiologically relevant for its cellular functions.

### NuMA Focuses MTs into Asters Together with Dynein

**Fig. 6.**
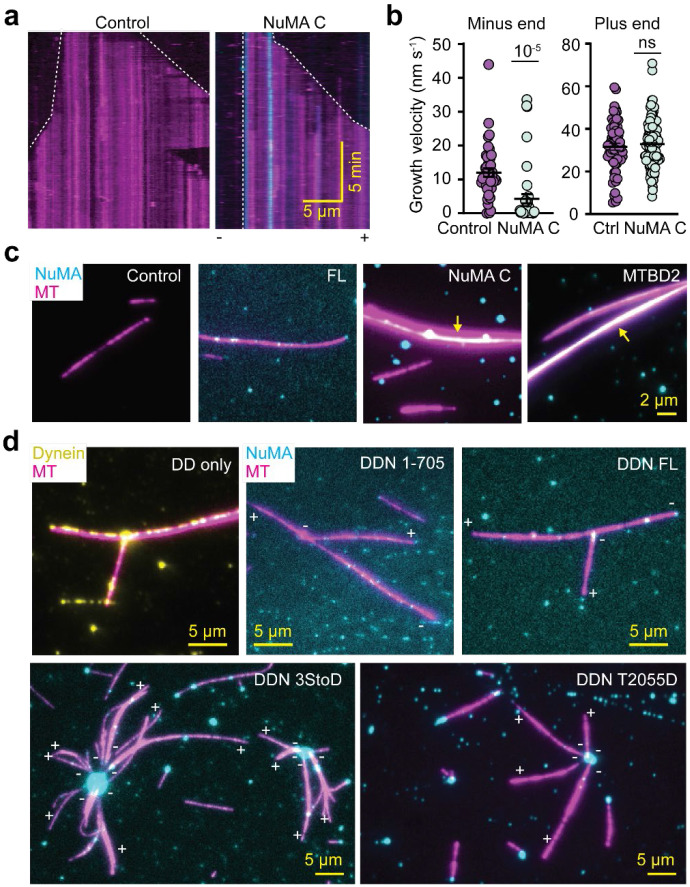
NuMA focuses MT minus-ends into asters with dynein/dynactin. **a.** Kymographs of dynamic MTs with GMPCPP MT seeds in the presence and absence of 100 nM NuMA C. Minus-end accumulation of NuMA C stops its growth and shrinkage. **b.** Growth velocities of the MT plus and minus ends in the absence and presence of NuMA C (n = 41, 43, 59, and 126 MT growth events from left to right). The centerline and whiskers represent mean and s.d., respectively. *P* values are calculated from a two-tailed t-test. **c.** Example pictures show that NuMA FL weakly interacts with freely diffusing MTs, whereas NuMA C and MTBD2 form MT bundles (yellow arrows). **d.** In the presence of dynein and dynactin (DD), NuMA T2055D and 3StoD focus MT minus-ends into aster-like structures. In comparison, DD alone, DDN with NuMA 1-705, and NuMA FL did not bundle or focus MTs. MT polarity is determined from the minus-end-directed motility of DDNs (not shown). The disappearance of the MT signal near the center of the asters in DDN 3StoD and T2055D conditions is due to the bending of MTs in the z direction near the NuMA cluster, which positions them away from the evanescent field of TIRF excitation. In a, c, and d, NuMA, MTs, and dynein are colored in cyan, magenta, and yellow, respectively.

We next investigated how NuMA affects the dynamics and organization of MTs with or without dynein. To determine whether MT binding of NuMA regulates MT dynamics, we immobilized MT seeds stabilized with a nonhydrolyzable GTP analog, GMP-CPP, to the glass surface and monitored active polymerization and depolymerization of MTs in the presence of free tubulin, GTP, and NuMA C (Fig. 6a). We observed NuMA C binding to both ends and the lattice of dynamic MTs. The accumulation of NuMA C at the MT minus-end almost completely stopped its growth, whereas there was little to no effect on the polymerization dynamics at the plus-end (Fig. 6b). These results show that the C terminal region of NuMA caps and stabilizes the minus-ends of MTs.

To investigate whether NuMA can crosslink and bundle MTs, we enabled the free movement of MTs while keeping them near the glass surface using the depletion forces generated by methylcellulose in the flow chamber. In the absence of NuMA, we observed little to no bundling of MTs (Fig. 6c). NuMA FL was bound to freely diffusing MTs but did not cause bundling, probably due to its low MT affinity. Although NuMA C or MTBD2 lack the coiled-coil region and do not form a dimer, they bundled the MTs to a significant extent, suggesting that the NuMA C terminus can simultaneously interact and crosslink multiple MTs.

**Extended Data Figure 9.**
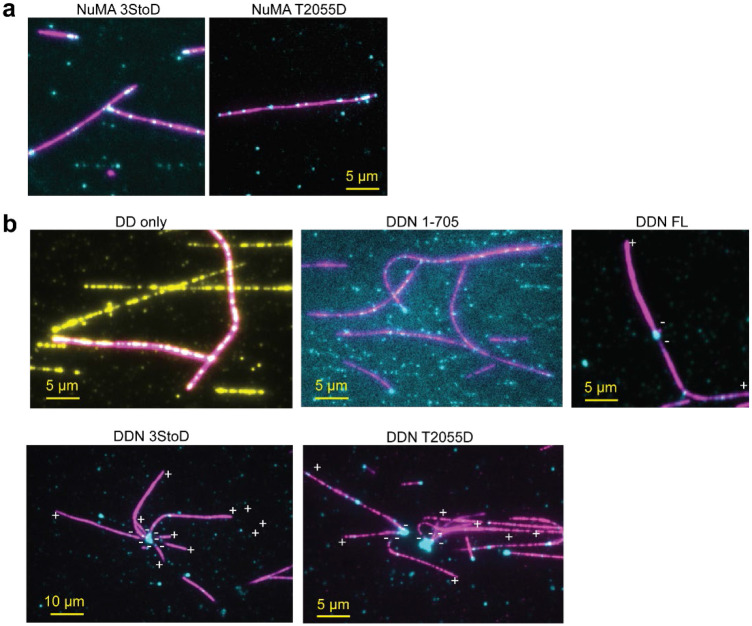
Additional examples of the organization of MTs into aster-like structures by DDN. **a.** NuMA 3StoD and T2055D bind to freely diffusing MTs but cannot bundle MTs or form MT asters. **b.** Additional examples of the organization of MTs into aster-like structures by active DDN complexes formed by NuMA 3StoD and T2055D. These structures were not observed in the absence of NuMA (DD only) or the presence of NuMA FL and NuMA 1-705. MT polarity was determined from the plus-end directed motility of Cy5-labeled K490 (not shown). Dynein, NuMA, and MTs are colored yellow, cyan, and magenta, respectively.

To test whether minus-end recognition and dynein activation of NuMA are sufficient to focus MTs into spindles, we mixed freely moving MTs and NuMA with or without dynein/dynactin and imaged their accumulation on surface-immobilized MTs (Fig. 6d, Extended Data Fig. 9). In the absence of dynein and dynactin, NuMA 3StoD or T2055D decorated the MT surface and occasionally caused bundling and coalescence of MTs, but we did not observe the formation of aster-like MT organization (Extended Data Fig. 9a). Similar results were observed for dynein and dynactin in the absence of NuMA (Fig. 6d). Remarkably, the addition of dynein, dynactin, and NuMA 3StoD or T2055D resulted in a robust assembly of MTs into asters (Fig. 6d, Supplementary Movie 4). During aster formation, typically, NuMA first appears as bright puncta at the minus-end of MTs. The coalescence of these MTs with another MT results in its minus-end directed transport by DDN. As a result, we observe a large accumulation of NuMA and focusing of MTs from their minus-ends at the center of the asters. Aster formation was not observed when we activated dynein motility with NuMA 1-705, which lacks the MTBDs. We concluded that both the MT-binding and crosslinking ability of NuMA are essential for dynein-mediated focusing of the minus-ends into aster-like structures.

### LGN Binding Favors the Interaction of NuMA with the Plus-end of MTs

**Extended Data Figure 10.**
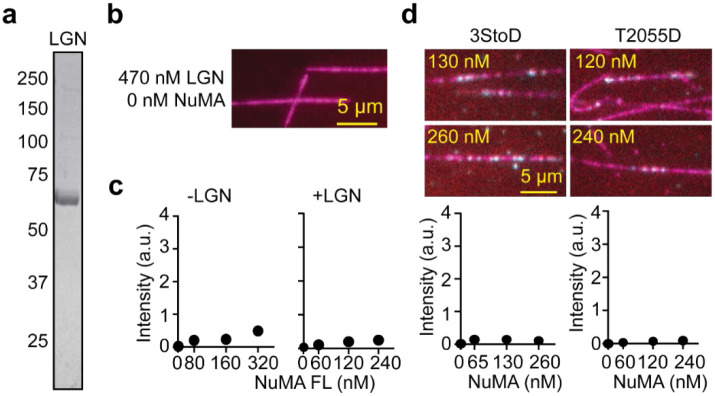
MT Binding of NuMA FL constructs in the presence of LGN. **a.** The denaturing gel picture of the LGN construct after gel filtration. **b.** LGN does not bind to MTs in the absence of NuMA. **c.** The intensity of NuMA FL per length of an MT in the presence and absence (from Fig. 4c) of 470 nM LGN (From left to right, n = 25, 67, 67, 116, 25, 90, 115, and 86 MTs). **d.** The intensity of 3StoD and T2055D per length of an MT in the presence of 470 nM LGN (From left to right, n = 25, 113, 110, 99, 25, 98, 95, and 138 MTs). In b and d, NuMA, LGN, and MTs are colored cyan, red, and magenta respectively. The white color represents the colocalization of all three proteins.

**Figure 7.**
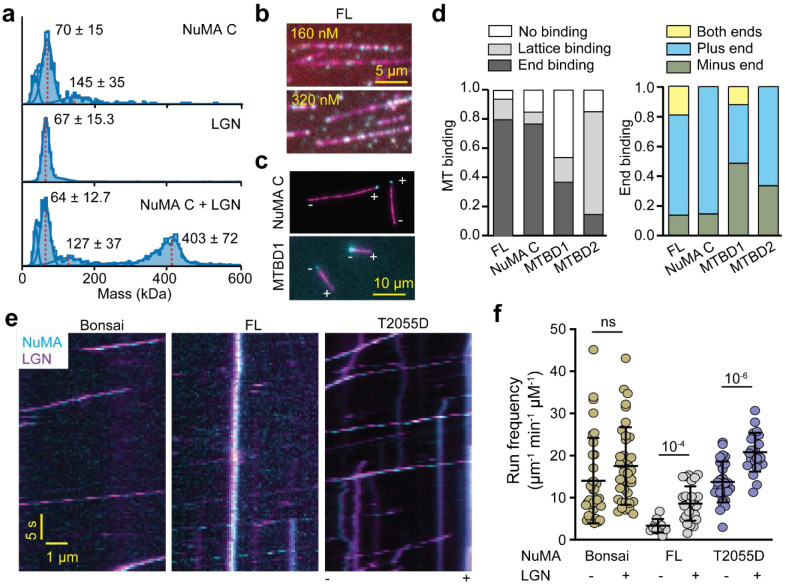
LGN favors MT plus-end binding and dynein activation of NuMA. **a.** Mass photometry shows that NuMA C and LGN are primarily monomers and form a 3:3 complex when mixed in equimolar (20 nM) concentrations. **b.** Example pictures show MT binding (magenta) of NuMA FL (cyan) in 470 nM LGN (red). The white color represents the colocalization of all three proteins. **c.** Example pictures show the colocalization of NuMA C terminal constructs (cyan) and 100 nM LGN (unlabeled) on MTs (magenta). MT polarity is determined by the plus-end-directed motility of K490 (not shown). **d.** Normalized MT binding and end binding preference of NuMA constructs in the presence of 100 nM LGN (n = 65, 73, 247, and 81 MTs from left to right). **e.** Kymographs show the comigration of LGN with DDN complexes on unlabeled MTs. **f.** The run frequency of DDN complexes in the presence and absence of LGN. The centerline and whiskers represent mean and s.d., respectively (n = 34, 38, 11, 29, 28, and 23 MTs from left to right). *P* values are calculated from a two-tailed t-test.

While the minus-end binding preference of NuMA is consistent with the accumulation and MT focusing of NuMA at the spindle poles, it remains unclear how NuMA binds to the plus-ends of astral MTs at the cell cortex (Fig. 1a). NuMA is recruited to the cortex by the interaction of its C-terminus with the N-terminal TPR domain of LGN^14, 60^. However, binding of LGN to NuMA is autoinhibited until its C-terminus binds to Gαi at the cell cortex^14, 60^. To investigate how LGN affects the MT binding of NuMA, we expressed the TPR domain of LGN (a.a. 1-409, LGN hereafter, Extended Data Fig. 10a), which readily binds to the NuMA C-terminus^14, 60^. Mass photometry confirmed that LGN forms a monomer (Fig. 7a). When mixed with NuMA C in equal amounts, we measured a mass population corresponding to the 3:3 LGN TPR and NuMA C complex (Fig. 7a), consistent with the crystal structure of the heterohexameric NuMA-LGN complex^24^.

We next investigated how LGN affects the MT binding of NuMA. As expected, LGN did not bind to MTs on its own, and it did not substantially affect the MT affinity of NuMA FL constructs (Extended Data Fig. 10b-d). In the presence of LGN, NuMA FL, NuMA C, and MTBD1 mostly bound to the tip of the MTs, whereas MTBD2 decorated the MT lattice (Fig. 7b-c). Although NuMA constructs exhibited preferential binding to the minus-end of MTs without LGN (Fig. 2), LGN binding to the NuMA C terminus resulted in preferential binding of NuMA FL and NuMA C to the plus-end of MTs (Fig. 7d). While MTBD1 maintained a slight preference to bind to the minus-end, MTBD2 preferred to bind to the plus-end of the MT when it only localizes to the tips of the MTs at low concentrations (Fig. 7d). These results raise the possibility that cortical recruitment of NuMA by LGN favors NuMA’s interaction with the plus-ends of astral MTs.

We also tested how LGN affects the ability of NuMA to activate dynein. Single-molecule motility assays showed that LGN co-migrates with virtually all processive DDN complexes assembled using NuMA constructs that contain both the N-terminal and C-terminal regions (Fig. 7e-f). The colocalization of LGN did not substantially affect the velocity or run frequency of the DDN Bonsai complexes. However, LGN binding increased the run frequency of DDN FL complexes (Fig. 7e-f), suggesting that LGN binding to the C-terminus partially rescues the autoinhibition of NuMA and favors the assembly of DDN complexes.

## Discussion

We used biochemical reconstitution with purified components and single particle cryo-EM and light microscopy to directly observe how NuMA activates dynein motility, binds MTs, affects MT dynamics, and organizes the MT filaments together with dynein *in vitro*. We showed that the N-terminus of NuMA forms a processive DDN complex together with dynein and dynactin and triggers dynein-driven transport when targeted to intracellular cargos in live cells. The C-terminal region of NuMA recognizes the minus-end of MTs through MTBD1 and suppresses its dynamics. The C-terminal region also binds to the MT lattice through MTBD2, which enables crosslinking of MTs. NuMA FL is autoinhibited and its ability to interact with MTs and dynein is significantly enhanced by phosphomimetic mutations to CDK1 and Aurora A phosphorylation sites at its C terminus. Together with dynein/dynactin, activated NuMA FL was able to sort and focus the minus-ends of MTs into aster-like structures reminiscent of spindle pole focusing of MT minus-ends during prometaphase^4^.

A puzzling feature of NuMA activation of dynein was the presence of both a Hook domain and a CC1-box-like motif. Our findings suggest that the CC1-box-like motif does not engage with DLIC, implicating the Hook domain as the primary mediator of this interaction. While we cannot exclude that the CC1-box-like motif interacts with dynein in other cellular contexts, our observation that the Hook domain is the only one relevant for the assembly of the full DDN complex is consistent with cellular observations^37^. Our structure also indicates that NuMA binds dynein and dynactin through unique Spindly motif and HBS1 sequences. These results shed light on the diverse mechanisms by which dynein and adaptors interact, suggesting a flexible, functionally specific framework for these interactions.

In interphase cells, NuMA is localized to the nucleus and unable to decorate MTs or interact with dynein. Our results show that interphase NuMA is inhibited for its mitotic functions and provide molecular insights into the autoinhibition and activation of NuMA during mitosis. We observed that the N-terminal Hook domain and the part of the coiled-coil (1-505 and 1-705) were most effective in the activation of dynein motility, whereas the inclusion of the rest of the coiled-coil or the C-terminus disfavored the assembly of DDN complexes. NuMA FL exhibits minimal dynein motility, suggesting that the interaction of NuMA with dynein is autoinhibited by the long coiled-coil and the NuMA C-terminus. Similarly, MTBD2 alone has a higher MT affinity than NuMA C and NuMA FL only weakly interacts with the MTs. The MT binding of NuMA may be partially autoinhibited by self-interactions within the C-terminal region, which may block the MTBD2 site and favor the minus-end recognition of the NuMA C-terminus. These results are also consistent with our previous observation that the coiled-coil domain prevents NuMA from binding chromosomes at mitosis^3^.

The autoinhibitory interactions may play a major role in repurposing NuMA for its diverse array of cellular functions. Our NuMA FL may represent NuMA’s interphase state, which does not strongly interact with MTs and dynein. In comparison, the assembly of NuMA with dynein and dynactin was activated by introducing phosphomimetic mutants of CDK1 and Aurora A phosphorylation sites. These results indicate that mitotic phosphorylation of NuMA does not simply regulate NuMA localization, but it is required to activate motility and force generation of dynein for spindle pole focusing and cortical force generation. While the underlying mechanism remains unclear, these phosphorylation events may disrupt some of the autoinhibitory interactions between the N- and C-termini of NuMA and allow NuMA to attain more open conformation to interact with its partners. In addition, these phosphorylation events also overlap with MTBD1 and MTBD2 sites and may alter the way NuMA interacts with the MT.

Our results also indicate that LGN binding to the NuMA C-terminus may repurpose NuMA for its cortical functions. We observed that LGN binding to NuMA enhances DDN motility, analogous to the activation of other dynein adaptors after they attach to their cargos^61^. LGN also favored the binding of NuMA to the MT plus-end, consistent with the interaction of LGN-bound NuMA with the plus-end tips of astral MTs at the cell cortex^1^. Because LGN binding partially overlaps with MTBD1^1^, it may disrupt the minus-end recognition of MTBD1, favoring the localization of NuMA to the plus-end tips of astral MTs. Plus-end recognition of the NuMA C-terminus may enable DDN motor complexes to capture the growing end of astral MTs and collectively pull the mitotic spindle under larger tension. Future studies are required to reveal whether NuMA can attain different conformational states through phosphorylation/dephosphorylation and interacting with binding partners for its specific functions.

Our results are largely consistent with an emergent *in vitro* study of the activation of dynein motility and the MT minus-end recognition of NuMA^62^. Unlike our observation that full-length NuMA is autoinhibited, Colombo et al. observed more robust MT interaction and dynein activation of NuMA FL^62^. Discrepancies between these studies may be relevant to differences in construct design and clustering of NuMA, or the usage of nonionic detergent to solubilize NuMA FL^62^, which may potentially affect autoinhibitory interactions.

NuMA is a unique dynein adaptor that interacts with MTs at its “cargo binding end”, thereby playing a central role in focusing the minus-ends of MTs^26, 50^. In spindle poles, most MTs are not directly anchored to the centrosomes^2^ but held together by NuMA^4, 9, 50, 63^. Because spindle poles recruit other MT motors, capping proteins, and crosslinkers, it is challenging to dissect the precise role of NuMA and dynein in the minus-end focusing of MTs. We demonstrated that NuMA and dynein/dynactin can efficiently focus minus-ends of MTs in the absence of other proteins. Aster formation requires the accumulation of NuMA at the MT minus-end, consistent with the ability of NuMA to localize to the spindle poles, albeit less efficiently, in the absence of dynein^9^. Transport of these MTs by DDN complexes as cargo towards the minus-end of another MT results in focusing and aligning of MT minus-ends into asters with a large accumulation of NuMA at its center. *In vivo* studies showed that focusing MTs into spindle poles also requires minus-end-directed kinesin-14, KIFC1 (HSET)^50^. KIFC1 is a weak, nonprocessive motor that localizes throughout the spindle MTs and is enriched at spindle poles^64-66^. KIFC1 contains a secondary nonmotor MT binding site, which facilitates crosslinking and aligning spindle MTs and inward sliding of these MTs against outward forces generated by kinesin-5s^67, 68^. In comparison, DDN is a processive motor that carries MTs toward the spindle poles and localizes to the extreme terminus of MT minus-ends^9, 50^. How these two minus-end-directed motors synergistically organize spindle MTs remains to be determined. The *in vitro* reconstitution assay we developed is poised to provide molecular insight into the specific contributions of MT motors, crosslinkers, and adaptors.

## Materials and Methods

### Cloning and plasmid generation

The plasmid pTK47_mCherry-LGN-Hardened was a gift from I. Cheeseman (Addgene plasmid #46347). All human NuMA and LGN constructs were cloned into the pOmnibac vector, incorporating an N-terminal His6-ZZ tag followed by a TEV protease cleavage site for protein expression and purification and a C-terminal SNAP or mNG sequence for fluorescent imaging. Full-length and C-terminal NuMA constructs were cloned into a vector containing an additional N-terminal MBP tag between the TEV cleavage and the ZZ tag to improve the solubility of expressed proteins. For live cell imaging, the coding sequence of NuMA-HaloTag-FRB constructs (NuMA 1-705, NuMA-Bonsai, NuMA 1-705 SpM, NuMA-FL, NuMA-FL-T2055D, and NuMA-FL-S1696D) were cloned into a puromycin-resistant lentiviral vector (Addgene #114021).

### Cell culture

hTERT-RPE1 cells were purchased from ATCC (CRL-4000). Lentivirus was produced in HEK293T cells that were purchased from ATCC (CRL-3216). All cells were grown in DMEM/F12 (ThermoFisher) supplemented with 10% tetracycline-screened FBS (PS-FB2; Peak Serum) and maintained at 37 °C and 5% CO_2_.

### Lentiviral plasmids, cell line constructions, and transfections

Lentivirus for each NuMA construct was produced in HEK293T cells. To generate stable polyclonal cell lines, wild-type RPE1 cells were infected with lentivirus and selected using 5 μg mL^−1^ puromycin. PEX3-mEmerald-FKBP (a gift from S. Reck-Peterson, UCSD) was transiently transfected into RPE1 cells to labeling peroxisomes for 2 or 3 days using Viafect (Promega) according to the manufacturer’s recommendations. Tubulin (including the centrosome) was labeled in cells by adding 100 nM SiR-tubulin and 10 μM verapamil for 60 min before imaging (Cytoskeleton). NuMA was labeled with 100 nM Janelia Fluor 549 (Promega) for 15 min before imaging. Rapamycin was added to a final concentration of 1 μM to induce dimerization of FRB-FKBP.

### Protein expression and purification

For baculovirus production, pOmnibac plasmids were transformed into DH10Bac competent cells and plated on Bacmid plates containing Bluo-Gal for 48 h at 37°C. Selected white colonies were grown overnight at 37°C in 2xYT medium. Bacmid DNA was purified using isopropanol precipitation and transfected into adherent SF9 cells (10^6^ cells mL^−1^) using FuGENE to produce P1 virus. The cells were cultured for 5–6 days at 27°C, and the P1 virus was harvested when more than 90% of the cells were transfected, as determined by monitoring fluorescent gene expression with a fluorescent light microscope. P2 virus was generated by infecting 50 ml of SF9 cells in suspension with 2 ml of P1 virus for 72 h at 27°C. The P2 virus was harvested by collecting the supernatant after centrifugation of SF9 cells at 4,000 g for 5 min at 4°C.

SF9 cells in 1 lt suspension were infected with 1% (v/v) P2 virus and cultured for 72 h at 27°C. The cells were collected by centrifuging at 4,000 g for 10 minutes and washed with ice-cold PBS before being flash-frozen for further purification. Cell pellets were lysed in a lysis buffer supplemented with 1 mM DTT, 2 mM PMSF, and two cOmplete protease inhibitor cocktail tablets (Roche) per liter of cells using a Dounce homogenizer. The lysate was clarified by centrifugation at 65,000 g for 30 minutes, and the supernatant was incubated with IgG Sepharose beads (Cytiva) for 1 hour at 4°C on a rotator. The beads were loaded onto a gravity-flow column and sequentially washed with a lysis buffer, followed by a wash buffer. The beads-protein mixture was then incubated with 0.1 mg/ml TEV protease overnight at 4°C. Proteins were eluted from the column using gravity flow and concentrated with molecular weight cutoff (MWCO) concentrators (Amicon). Protein concentration was determined by measuring absorbance at 280 nm using a NanoDrop 1000 (Thermo Fisher). Proteins containing a SNAP tag were fluorescently labeled by incubating them with a 5-fold molar excess of BG-LD655 or LD555 dye (Lumidyne). Excess dye was removed by gel filtration.

Dynein-expressing cell pellets were lysed in 50 mM HEPES (pH 7.4), 100 mM NaCl, 2 mM MgCl_2_, 0.1 mM ATP, and 10% glycerol. The beads-protein mixture was washed with a buffer containing 50 mM Tris-HCl (pH 7.4), 150 mM KAc, 2 mM Mg(Ac)_2_, 1 mM EGTA, 0.1 mM ATP, and 10% glycerol. The protein was then loaded onto a TSKgel G4000SWXL size exclusion column (Tosoh) for further purification.

KIF5B (1-490) was purified using a lysis buffer consisting of 50 mM HEPES (pH 7.4), 1 M NaCl, 1 mM DTT, 2 mM PMSF, 2 mM MgCl_2_, 0.1 mM ATP, and 10% glycerol. The wash buffer contained 50 mM HEPES (pH 7.4), 300 mM NaCl, 1 mM DTT, 2 mM MgCl_2_, 0.1 mM ATP, and 10% glycerol.

Cell pellets expressing NuMA N-terminal constructs were lysed and washed with 50 mM HEPES (pH 7.4), 300 mM NaCl, 1 mM EGTA, and 10% glycerol. The lysate was loaded onto a Superose 6 Increase 10/300 GL (Cytiva) column for size exclusion chromatography in a buffer containing 50 mM HEPES (pH 7.4), 150 mM NaCl, 1 mM EGTA, and 10% glycerol.

For FL NuMA protein purification, cells were lysed in 50 mM HEPES (pH 7.4), 30 mM NaCl, 2 mM MgCl_2_, 1 mM EGTA, and 10% glycerol, supplemented with benzonase nuclease (Sigma). The NaCl concentration was then increased to 300 mM before centrifugation. The FL NuMA protein-beads mixture was washed with 50 mM HEPES (pH 7.4), 300 mM NaCl, 1 mM EGTA, and 10% glycerol and loaded onto a Superose 6 Increase 10/300 GL (Cytiva) column for size exclusion chromatography in 50 mM HEPES (pH 7.4), 150 mM NaCl, 1 mM EGTA, and 10% glycerol.

Cells expressing NuMA C-terminal constructs were lysed in 50 mM HEPES (pH 7.4), 1 M KCl, 1 mM EGTA, and 10% glycerol. The lysate was washed with 50 mM HEPES (pH 7.4), 300 mM KCl, 1 mM EGTA, and 10% glycerol and loaded onto a Superdex 200 Increase 10/300 GL (Cytiva) column for size exclusion chromatography in 50 mM HEPES (pH 7.4), 150 mM KCl, 1 mM EGTA, and 10% glycerol.

For LGN purification, cells were lysed in 100 mM Tris-HCl (pH 8.0), 300 mM NaCl, 0.5 mM EDTA, and 5% glycerol. The eluted protein from the beads was loaded onto a Resource-Q ion exchange column using a desalting buffer containing 20 mM Tris-HCl (pH 8.0), 40 mM NaCl, 0.5 mM EDTA, and 5% glycerol. LGN bound to the ion exchange column was eluted using a salt gradient from 40 mM to 450 mM NaCl over 20 column volumes.

Mammalian dynactin was purified as previously described from pig brains using SP Sepharose Fast Flow, MonoQ ion exchange columns (Cytiva), and the TSKgel G4000SWXL size exclusion column (Tosoh)^45^.

### Cryo-EM sample preparation

To prepare MTs, porcine brain tubulin was diluted in BRB80 buffer (80 mM PIPES pH 6.8, 1 mM MgCl_2_, 1 mM EGTA) to a final concentration of 6 μM. GMPCPP was added to a final concentration of 1 mM and polymerization was induced by incubating at 37 °C for 1 h. MTs were pelleted by centrifugation at 20,000 *g* for 8 minutes and resuspended in BRB80, after which MTs were depolymerized by incubating 30 min on ice. A second polymerization round was performed by adding GMPCPP to a final concentration of 1 mM and incubating the solution at 37 °C for 1 h, followed by another round of centrifugation and resuspension in BRB80. Bradford assay (BioRad) was used to estimate the protein concentration, and MTs were diluted to 0.3 mg mL^−1^ immediately before use.

The DDN 1-705 complex was assembled by mixing the purified dynein, dynactin, Lis1, and NuMA in a 1:2:50:50 molar ratio, respectively, in GF150 buffer (25 mM HEPES pH 7.2, 150 mM KCl, 1 mM MgCl_2_, 1 mM DTT), with dynein at a final concentration of 0.14 μM in 5 μL. The assembly solution was incubated on ice for 30 min and subsequently diluted 2-fold to a final volume of 10 μL in GF150. To bind the complex to MTs, 9 μL of the complex was mixed with 5 μL of diluted MTs and 9 μL of S1 buffer (25 mM HEPES pH 7.2, 5 mM MgSO_4_, 1 mM EGTA, 2 mM DTT, 25 mM AMPPNP) and incubated at room temperature for 15 min. MT-bound complexes were separated from unbound protein by pelleting at 20,000 g for 8 min, and then resuspended in 80 μL S2 buffer (25 mM HEPES pH 7.2, 35 mM KCl, 5 mM MgSO_4_, 1 mM EGTA, 1 mM DTT, 5.3 mM AMPPNP, 0.01% IGEPAL (Millipore-Sigma)). 4 μL of resuspended solution was applied to freshly glow-discharged Quantifoil R2/2 mesh 300 gold grids (Quantifoil) in a Vitrobot IV (ThermoFisher) at 100% humidity and 22 °C, incubated for 45 s, and blotted for 1.5 s at a blot force of −15 before plunge-freezing into liquid ethane.

### Cryo-EM data acquisition

The sample was imaged using a FEI Titan Krios (300 kV) equipped with a K3 detector and energy filter (20 eV slit size, Gatan) using automated data collection (ThermoFisher EPU). A total of 44,287 movies was acquired at 81,000X magnification (1.059 Å/pixel), with a 2.32 s exposure time, 40 fractions, and fluence of ~40 e^−^/Å^2^. A 100 μm objective aperture and a defocus range between −1.2 and −2.2 μm were used during image acquisition.

### Cryo-EM image processing

Global motion correction and the contrast transfer function (CTF) estimation were performed using MotionCor2 and CTFFIND4 within RELION 4.0 respectively^69-71^. MTs were initially picked using crYOLO 1.9^72^ in single particle mode, utilizing a model trained on manually picked micrographs in filament mode. The coordinates were then resampled at a 4 nm interval using a multi-curve fitting script, resampled coordinates were split into short segments of 10 coordinates each, and MT subtraction was subsequently performed, as previously described^73^.

The resulting "once-subtracted" micrographs were fed back into crYOLO for a second, third, and fourth round of MT picking, multi-curve fitting, filament splitting, and subtraction, all using the same parameters as the first round. After the fourth round, the quadruple-subtracted micrographs were utilized to pick complexes using crYOLO. If no MTs were detected following a given round of subtraction, the last successfully subtracted micrograph was used instead for particle picking.

The picked particles were extracted using RELION 4.0 and imported into CryoSPARC^74^, for particle sorting. Each step of sorting was repeated multiple times to minimize the effects of stochastic variations in sorting results, with the best class of all parallel iterations pooled together. Firstly, particles went through a round of heterogeneous refinement using eight reference volumes, out of which six were contaminants, one was a dynein-dynactin-BICD2 complex with two dynein dimers, and one was a dynein-dynactin-BICD2 complex with one dynein dimer. These volumes were previously determined to be effective in isolating *bona fide* dynein-dynactin-adaptor complexes (unpublished results). The one-dynein class degraded during refinement, so only the two-dynein class was taken forward. Heterogeneous refinement was followed by two rounds of 3D classification in cryoSPARC, with masks spanning the entirety of the complex first, and the more stable component afterwards. In each round, the class with the best density for dynein-dynactin-adaptor was taken forward. The particles coming out of the last classification were locally refined and exported into RELION 5.0 for particle polishing and rebinning, after which they were imported again into CryoSPARC for CTF refinement and particle subtraction. A graphical summary of the processing pipeline including particle numbers, FSCs, B-factors, intermediate maps, and masks used for refinements, classifications, and particle subtractions are shown in Extended Data Figure 2.

### Model building and refinement

Model building was performed using ISOLDE^75^ and COOT^76^, and refinements were performed in PHENIX^77^. Protein Data Bank (PDB) models or AF2 predictions were docked into cryo-EM maps using UCSF ChimeraX^78^. Refinement statistics are available in Extended Data Table 2. AF2 models were generated using a local installation of ColabFold 5^79^, using MMSeq^80^ for homology searches and AlphaFold2-Multimer^81^.

To build the full complex model, we docked coiled-coil fragments from an AF2 model of NuMA (1-705) dimer into the adaptor density based on their length and fitted using restrained flexible fitting in ISOLDE. We could not model the linkers between coiled-coil fragments. We have assigned fragments together based on distance, but we could not exclude that the other possible arrangement might be accurate. The model of dynactin and the dynein tails was built using PDB ID 8PTK^44^ as the starting model. To model the CC3 fragment, including the Spindly motif and the CC1b-CC2 linker, we used an AF2 model of NuMA (351-450) dimer and the pointed end subunits, which was joined to the CC1b and flexibly fitted with restraints into the density. Using the density from the pointed end subcomplex local refinement, we could assign the CC2 making the Spindly box contacts and the CC1b contacting the side of the pointed end to the same molecule. The linker connecting the other protomer was not resolved and deleted at this stage.

### Negative Stain EM

Proteins for negative stain were diluted to 20 nM with the following buffer (50 mM HEPES pH 7.4, 150 mM KCl) and 4 μL of sample was applied to homemade carbon-coated grids after glow discharging the grids. Samples were then stained with 1.5% uranyl formate, blotted, and then air dried. Imaging was performed at ~30,000x magnification with a Tecnai 12 microscope.

### Fluorescence Microscopy

For *in vitro* imaging, a custom-built multicolor objective-type TIRF microscope was used, equipped with a Nikon inverted Ti-E microscope body, a 100X magnification, 1.49 N.A. apochromatic oil-immersion objective (Nikon), and a Perfect Focus System. Fluorescent signals were detected using an electron-multiplied charge-coupled device (EMCCD) camera (Andor, iXon EM+, 512 × 512 pixels), resulting in an effective pixel size of 160 nm after magnification. The mNG, LD555, and LD655 probes were excited by 488 nm, 561 nm, and 633 nm laser beams (Coherent), respectively. The fluorescence signal was filtered using a notch dichroic filter and 525/40, 585/40, and 695/75 bandpass emission filters (Semrock) for the respective probes. Micro-Manager 1.4 was used to control the microscope and acquire movies.

For live cell imaging, cells were plated onto #1.5 glass-bottom 35 mm dishes coated with poly-D-lysine (P35G-1.5-20-C, MatTek Life Sciences) and imaged in a humidified stage-top incubator maintained at 37 °C and 5% CO_2_ (Tokai Hit). Cells were imaged on a spinning disk (CSU-X1, Yokogawa) confocal inverted microscope (Eclipse Ti-E, Nikon Instruments) with the following components: Di01-T405/488/568/647 head dichroic (Semrock); 488 nm (120 mW), 561 nm (100 mW), and 639 nm (160 mW) diode lasers; ET525/36M, ET600/50M, and ET676/37M emission filters (Chroma Technology); and an iXon3 Ultra 897 EMCCD camera (Andor Technology). Images were acquired with a 100x 1.45 Ph3 oil objective (Nikon Instruments) using Micromanager 2.0.0.

### Flow chamber preparation

For *in vitro* fluorescence imaging assays, polyethylene glycol (PEG)-coated coverslips were used to prevent nonspecific surface binding of proteins. The coverslips were washed with water, acetone, and water again, each for 10 min in an ultrasonic cleaner. For hydroxylation, they were incubated in 1 M KOH for 40 min in the ultrasonic cleaner, followed by rinsing with water and methanol. The coverslips were then treated with an aminosilanization mixture containing 3-aminopropyltriethoxysilane, acetate, and methanol for 10 min, rinsed with water for 1 min, and subjected to a second 10 min incubation in the aminosilanization mixture in the ultrasonic cleaner. The coverslips were then rinsed with methanol and air-dried. Hydroxylated coverslips were biotinylated by placing 30 μl of 25% biotin-PEG-succinimidyl valerate in NaHCO_3_ buffer (pH 7.4) between two coverslip pieces, forming a sandwich, and incubating overnight at 4°C. The coverslips were then rinsed with water, air-dried, vacuum-sealed, and stored at −20°C. Flow chambers were prepared by attaching the PEG-biotin-coated coverslip to a glass slide using double-sided tape.

### MT polymerization assay

Biotinylated MTs were prepared by diluting 4 μl of 38 mg/mL unlabeled pig brain tubulin and 2 mg/ml biotin-labeled pig brain tubulin in 56 μl BRB80 buffer (80 mM PIPES, pH 6.8, 1 mM MgCl_2_, 1 mM EGTA). This mixture was combined with 60 μl of polymerization buffer (1x BRB80, 2 mM GTP, 20% DMSO) and incubated at 37°C for 45 min. After adding 1 mM taxol, the tubulin mixture was incubated for an additional 45 min at 37°C. The polymerized MTs were pelleted by centrifugation at 21,000 g for 15 min at 37°C and resuspended in 25 μl of BRB80 supplemented with 10 μM taxol and 1 mM DTT. The same procedure was followed to prepare biotin-free, fluorescently labeled MTs by diluting 4 μl of 40 mg/mL pig brain tubulin, 5% of which was Cy3- or Cy5-labeled. For subtilisin treatment, 2 mg/mL polymerized MTs were incubated with 0.025 mg/mL subtilisin for 1 h at 37° C. The proteolytic activity of subtilisin was quenched by adding 1 mM PMSF. The cleavage of the tubulin tails by subtilisin was confirmed using denaturing gel analysis.

For GMPCPP-MT seed preparation, a mixture of unlabeled tubulin, 5% biotinylated tubulin, and 5% Cy3-labeled tubulin was diluted to 3 mg/mL in ice-cold BRB80 containing 10% DMSO. After 3 min incubation on ice, the mixture was centrifuged at 400,000 g for 10 min at 4°C. The supernatant was mixed with GMPCPP to a final concentration of 1 mM and incubated at 37°C for 20 min. The GMPCPP-stabilized MT seeds were pelleted by centrifugation at 400,000 g for 10 min at 37°C. The pellet was gently resuspended in 25 μL of BRB80 at room temperature.

### Single-molecule motility assays

To immobilize biotinylated MTs, the PEG/PEG-biotin coated flow chambers were incubated with 1 mg/ml streptavidin for 3 min, followed by a wash with MB buffer (30 mM HEPES pH 7.4, 5 mM MgSO_4_, 1 mM EGTA, 1 mM DTT, and 10 μM Taxol). The biotinylated MTs were then flowed into the chamber, incubated for another 3 min, and washed with MB buffer. For dynein motility assays, dynein and dynactin were mixed and incubated on ice for 3 min. NuMA was then added and incubated for an additional 3 min, followed by the addition of Lis1 with a further 3 min incubation. The molar ratio of the DDNL components in the stock mixture was 1:3:20:30 (dynein:dynactin:NuMA:Lis1) for N terminal NuMA constructs and 1:3:5:40 for NuMA FL constructs. The final volume of the DDNL stock mixture was adjusted to 10 μL by adding MBC buffer (MB buffer supplemented with 1 mg/mL casein, 0.5% pluronic acid, 100 mM KAc, and 0.4% methylcellulose). The DDNL stock was diluted 10-fold for the FL NuMA construct and 60-fold for the N-terminal NuMA construct. Both dilutions were prepared in imaging buffer (MBC buffer supplemented with 0.1 mg/mL glucose oxidase, 0.2 mg/mL catalase, 0.8% D-glucose, and 1 mM ATP) and introduced into the flow chamber. For DDN motility assays with N-terminal NuMA, 20 nM NuMA and 30 nM Lis1 were used in the flow chamber. For assays with FL NuMA, 20 nM NuMA and 160 nM Lis1 were used. Motility was recorded for 200 frames with 0.2 s exposure per frame.

For aster formation assays, biotinylated MTs were immobilized to the PEG/PEG-biotin surface. The DDNL mix with Cy3-labeled, biotin-free MTs was then flowed into the chamber. The aster formation was imaged by overlaying the mNG-labeled NuMA and Cy3-labeled MT channels. To assess the directionality of the MTs, Cy5-labeled KIF5B (a.a. 1-490) was added to the channel and imaged.

### NuMA MT binding assays

To test the MT binding affinity of NuMA, taxol-stabilized MTs were labeled with Cy3 and biotin and immobilized on PEG/PEG-bio coated coverslips using streptavidin-biotin linkage. Serial dilutions of each mNG fused NuMA construct were flowed into the channel in MBC buffer supplemented with 0.1 mg/mL glucose oxidase, 0.2 mg/mL catalase, and 0.8% D-glucose. Images were taken for both the NuMA and MT channels under each condition. To study the effect of LGN on NuMA MT binding affinity, serial dilutions of NuMA constructs were initially mixed with LD655 labeled LGN for 3 min on ice before being flowed into the flow chamber.

To test the MT binding preference of NuMA, Cy3-labeled free-floating MTs were mixed with NuMA for 3 minutes, diluted in MBC buffer supplemented with 0.1 mg/mL glucose oxidase, 0.2 mg mL^−1^ catalase, 0.8% D-glucose, and 1mM ATP, and then flowed into a channel. 15 nM Cy5 labeled human kinesin-1 K490 was included in the imaging buffer to determine the directionality of the MTs. Final concentrations of 25 nM NuMA C, 165 nM MTBD1, 4 nM MTBD2, 25 nM NuMA-FL, 25 nM 3StoD, and 25 nM T2055D were used. To study the effect of LGN on NuMA MT binding preference, NuMA-mNG, unlabeled LGN (100 nM), and Cy3 labeled biotin-free MTs were incubated for 3 minutes, flowed into the channel, and imaged.

### Dynamic MT assays

Biotinylated GMPCPP-MT seeds were immobilized in a 1 mg/mL streptavidin-coated flow chamber by incubating for 3 min. To generate dynamically growing and shrinking MTs, a dynamic MT mixture (2 mg/mL tubulin with 5% Cy3 labeling, 4 mM GTP, 1 mM ATP, 1 mg/mL casein, 0.5% pluronic acid, 1 mM DTT, and 0.2% methylcellulose in BRB80, pH 6.8,) was introduced into the chamber. To assess its effect, 100 nM of the NuMA C-mNG was added to the mixture. MT dynamics was monitored by timelapse imaging with 200 frames, a 0.2 s exposure time, and a 5000 s interval between the frames.

### Mass photometry

High-precision coverslips (Azer Scientific) were cleaned sequentially with isopropanol and water three times, using an ultrasonic cleaner for 2 min per cycle, and then air-dried. Gaskets were rinsed alternately with water and isopropanol three times, air-dried, and placed on a clean coverslip. For autofocus, 14 μL of mass photometry buffer (50 mM HEPES, pH 7.4, 150 mM NaCl, 1 mM EGTA, and 10% glycerol) was loaded into a well of the gasket. The protein sample was diluted to 5–20 nM in mass photometry buffer and added to the autofocused gasket well. Protein contrast counts were measured using a TwoMP mass photometer (Refeyn 2) with a minimum of two replicates. The instrument was calibrated for mass measurements using a mixture of conalbumin, aldolase, and thyroglobulin. DiscoverMP software (Refeyn) was used to fit the mass photometry profiles to multiple skewed Gaussian peaks and to determine their mean, standard deviation, and proportions.

### Peroxisome Trafficking Assay

Cells expressing both PEX3-mEmerald-FKBP and a NuMA-HaloTag-FRB construct were imaged every 30 seconds for approximately 50 minutes. Rapamycin was added to the dishes after 90 seconds of imaging. At the end of the timelapse imaging, a z-stack was taken to determine whether peroxisomes had clustered at the centrosome. Peroxisome trafficking was determined from the z-stacks taken after approximately 50 minutes. We quantified PEX3 intensity (integrated density) at the centrosome (2.54 μm x 2.54 μm oval selection) using FIJI. The intensity was normalized to the cell background (average of three 2.54 μm x 2.54 μm oval selections in the cytoplasm at the same z-slice). A cell was considered to have trafficking if the normalized GFP intensity was > 2. We also quantified NuMA concentration (mean pixel value) in the cytoplasm (10.07 μm x 10.07 μm rectangle selection) using FIJI. Concentration was averaged over three timepoints before rapamycin addition and normalized to the mean of control. In timelapses where the centrosome remained in focus for the majority of imaging, we measured PEX intensity (again, integrated density in a 2.54 μm x 2.54 μm oval selection at the centrosome in FIJI) at every timepoint (Extended Data Fig. 8d) and normalized to the first timepoint of imaging (pre-rapamycin addition). Individual timepoints where the cell temporarily was out of focus, where out of focus was determined by looking at the centrosome, were excluded from the traces.

### Data analysis

Recorded movies of DDN single-molecule motility assays were analyzed using ImageJ. For two-color imaging, the fluorescence channels were merged in ImageJ to create a composite image. A custom ImageJ macro was used to generate kymographs by plotting segmented lines along MTs. For run frequency analysis, the number of processive NuMA molecules on each MT was divided by the MT length (μm), time (min), and NuMA concentration (μM). Dynein’s velocity and run length were determined by identifying the start and end points of each processive NuMA run. For MT binding assays, the intensity of NuMA on the MTs was quantified after background subtraction using a custom MatLab code (MTIMBS). The dynamic MT growth velocity was calculated by drawing kymographs and identifying the start and end points of each growing end in ImageJ.

To determine the binding preference of different NuMA constructs on biotin-free MTs, NuMA fused to mNG, Cy3-labeled MTs, and Cy5-labeled KIF5B (1-490) channels were overlaid. MTs with mNG signal accumulated at one or both ends were manually counted as exhibiting end-binding. The directionality of the MTs was confirmed by analyzing the movement of KIF5B (1-490) through kymographs. NuMA-bound MTs without signal accumulation at the tips were quantified as displaying lattice binding.

### Statistical analysis

*P* values were determined using statistical tests in Prism. Fisher’s exact tests were performed to compare categorical datasets. Two-sided two-sample t-tests were performed to compare continuous datasets, assuming that NuMA and dynein levels are approximately normally distributed. Each result was based on a minimum of two independent experiments. The number of replicates and data points (n) and the specific statistical methods used are provided in the figure legends. Representative data from independently repeated experiments are shown.

## Extended Data

**Extended Data Table 1.** Cryo-EM data collection and refinement statistics.

	DDNL consensus	Pointed end with NuMA
**EMDB ID**	52171	52172
**PDB ID**	9HHL	-
		
**Data collection**		
Voltage	300 kV	300 kV
Electron exposure	40 e^−^/Å^2^	40 e^−^/Å^2^
Magnification	81000x	81000x
Pixel size	1.059 Å/px	1.059 Å/px
Defocus range	−1.2 to −2.2 μm	−1.2 to −2.2 μm
Total micrographs	44,287	44,287
Initial particle images	1,418,923	1,418,923
Final particle images	8,092	8,092
Map resolution	6.53 Å	6.78 Å
FSC threshold	0.143	0.143
Sharpening B-factor	−202	−380
		
**Model refinement**		
Model Resolution	8.8	-
FSC threshold	0.5	-
		
**Model composition**		-
Non-hydrogen atoms	64693	-
Protein residues	13016	-
Ligands	Zn: 3 ADP: 8 ATP: 2	-
		
**RMSD deviations**		
Bond lengths	0.004 Å	-
Bond angles	1.190 °	-
		
**Validation**		
Clashscore	3.53	-
MolProbity score	1.33	-
Rotamers outliers (%)	0.00	-
		
**Ramachandran plot**		
Favored	96.87 %	-
Allowed	3.10 %	-
Outliers	0.03 %	-

## Supplementary Material

Supplement 1 **Supplementary Movie 1. The NuMA N terminus activates dynein motility.** One color TIRF imaging of representative DDN N-terminal complexes on surface-immobilized unlabeled MTs in the presence of Lis1. NuMA was labeled with LD555, and the images were acquired at 200 ms exposure time per frame.

Supplement 2 **Supplementary Movie 2. FL NuMA is autoinhibited and phosphorylation through its C-terminus activates DDN motility.** Single color TIRF imaging of DDN FL complexes on surface-immobilized unlabeled MTs in the presence of Lis1. The motility was monitored by the fluorescence signal of NuMA-mNG at 200 ms exposure time per frame.

Supplement 3 **Supplementary Movie 3. NuMA can activate dynein in interphase cells.** Timelapse confocal imaging of representative WT RPE1 control cell, transfected with PEX3-mEmerald-FKBP (grey), infected or transfected with various NuMA-HaloTag-FRB constructs (not shown), and stained with SiR-tubulin (not shown), with rapamycin addition at time 0:00 (min:s). Peroxisomes randomly diffuse throughout the cytoplasm in Control and in conditions where the NuMA construct does not activate dynein. Peroxisomes traffic to the centrosome, as defined by SiR-tubulin staining, when the NuMA construct activates dynein. Individual cells come from various days, and so PEX3-mEmerald-FKBP levels vary between cells due to variations in transfections. The movie corresponds to still images in Figure 5b. Scale bar = 5 μm.

Supplement 4 **Supplementary Movie 4. Active NuMA FL forms asters by focusing the minus-ends of MTs with dynein/dynactin.** Two-color imaging of DDN 3StoD motility on surface-immobilized unlabeled MTs in the presence of free-floating Cy3 labeled MTs (magenta) and Lis1. The motility was monitored by the fluorescence signal of NuMA-mNG (cyan). The images were acquired at 200 ms exposure time per frame.

## Data Availability

A reporting summary for this article is available as the Supplementary Information file. The main data supporting the findings of this study are available within the article and its Extended Data Figures. Protocols that support the findings of this study can be found in Methods. The constructs that express wild-type and mutant versions of NuMA will be deposited to AddGene. Raw microscopy data will be made available by the corresponding authors upon request. Cryo-EM structures will be deposited to PDB and EMDB.

## Code Availability

The custom code used to analyze experimental data is uploaded to the Yildiz Lab code repository (www.yildizlab.org/code_repository) and GitHub (https://github.com/Yildiz-Lab/MTIMBS).
